# Supplementary material for: PARP1 allows proper telomere replication through TRF1 poly (ADP-ribosyl)ation and helicase recruitment
Source: Commun Biol. 2023 Mar 2;6:234. doi: 10.1038/s42003-023-04596-6 (PMC9981704; doi:10.1038/s42003-023-04596-6)

Supplemental Figure 1

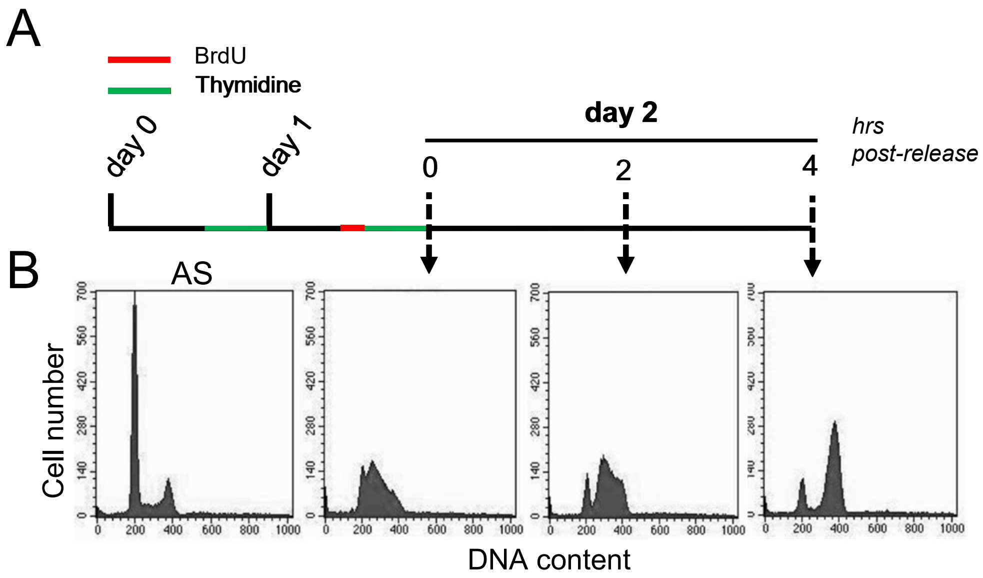

Supplemental Figure 1. Scheme and analysis of cell synchronization in S-phase. Hela cells were pulsed twice with thymidine. A BrdU pulse of 15 minutes was administered before the second pulse (**A**). Then samples were released in fresh medium and collected at the indicated time points. Samples were then processed for cytofluorimetric analysis of DNA content (**B**).

Supplemental Figure 2

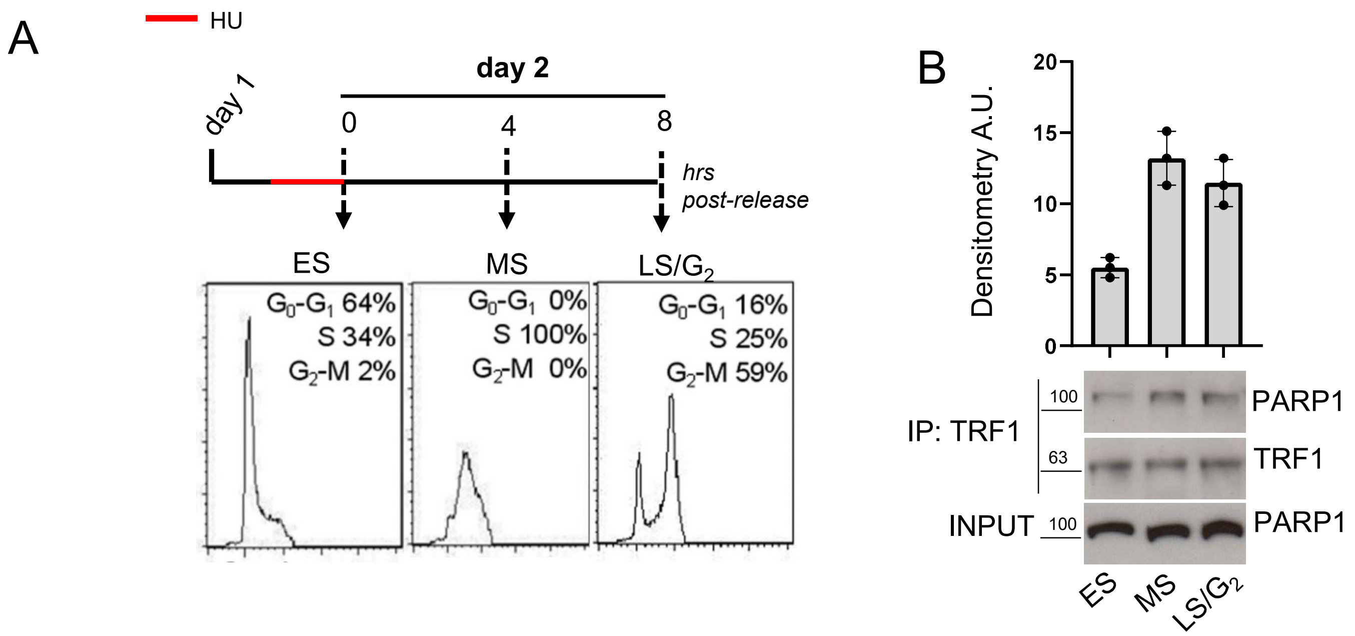

Supplemental Figure 2. PARP1 and TRF1 affinity increases during S-phase. BJ EHLT cells were synchronized at the G1-S boundary with 0,5 mM Hydroxyurea for 16 hours. Then samples were released in fresh medium and collected at the indicated time points. Samples were then splitted and processed for cytofluorimetric analysis of DNA content (**A**) or for immunoprecipitation with anti-TRF1 (Santa Cruz) specific antibody (**B**) and finally incubated with anti-PARP1 (BD). Histograms in **B** report the quantification of PARP1 signals in immunoprecipitated samples normalized on PARP1 content in the input. TRF1 levels in the input are shown as loading controls.

Supplemental Figure 3

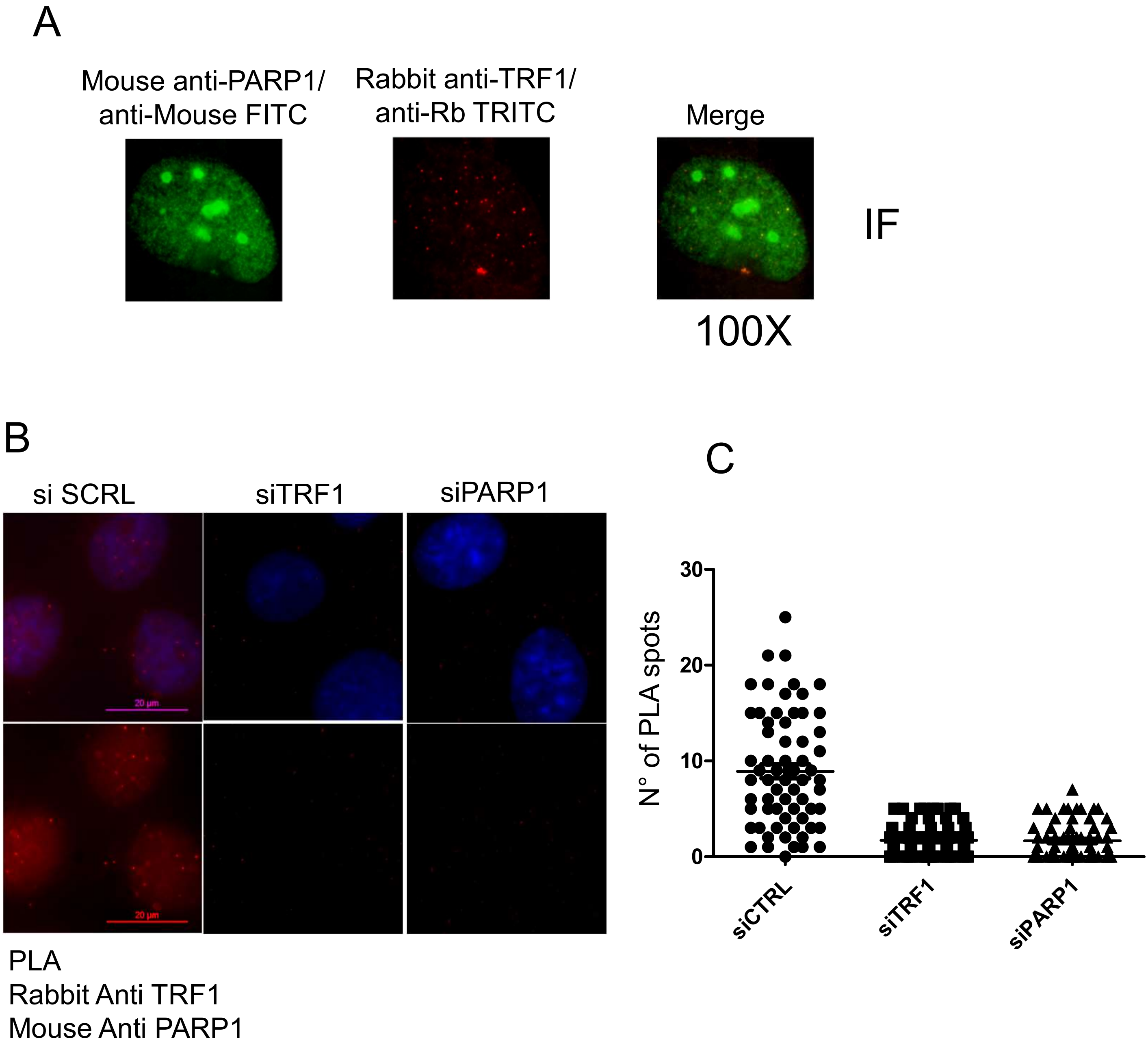

Supplemental Figure 3. A: Hela cells were seeded, fixed and processed for co-immunofluorescence with anti-mouse PARP1 and anti-rabbitTRF1 specific antibodies followed by the indicated secondary antibody. Fluorescent signals corresponding to both proteins staining are shown in representative images at 100X magnification. **B**: cells transfected with the indicated siRNAs were processed for co-IF with the above primary antibodies and processed for Proximity Ligation Assay with the DUOLink Red kit mouse/rabbit (Sigma). Representative images at 63X magnification are shown. **C:** Quantification of the PLA spots is shown in the graph in the right panel.

Supplemental Figure 4

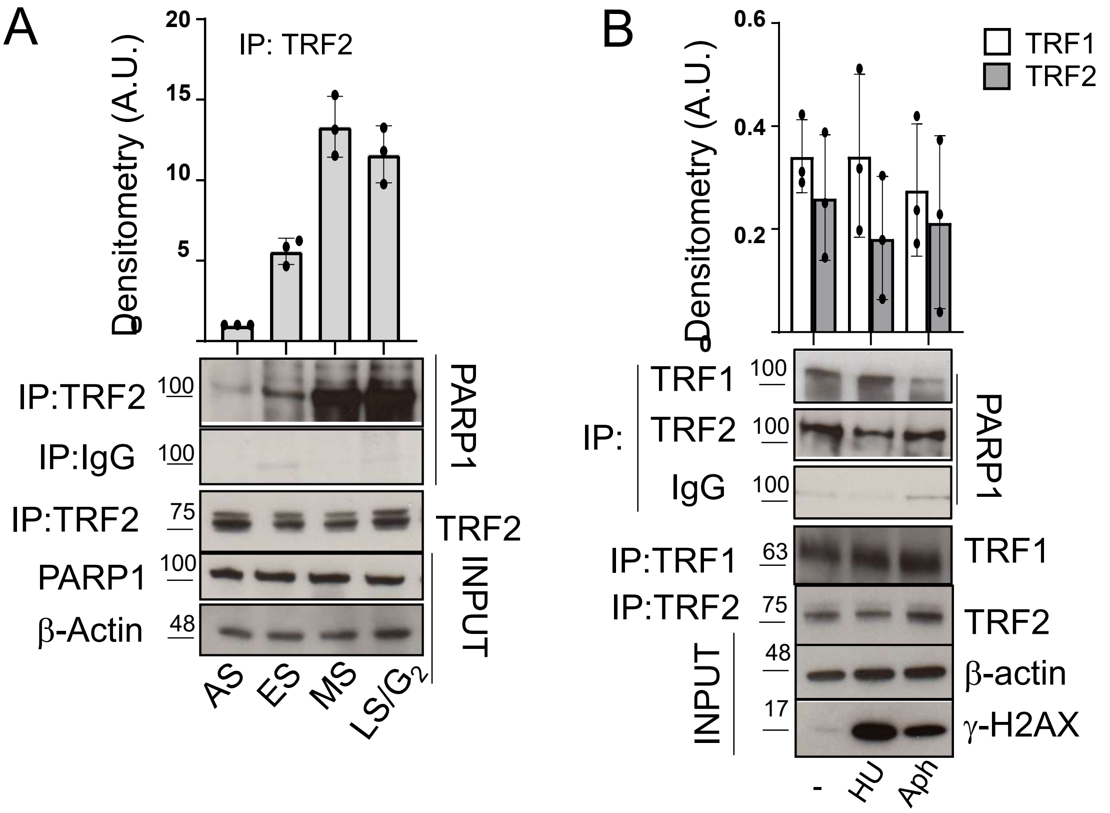

Supplemental Figure 4 A: TRF2 and PARP1 interacts during S-G$_2$M. Hela cells were synchronized in the early S phase by double thymidine block and then released and collected at the indicated time points. Samples collected underwent immunoprecipitation with an anti-TRF2 specific antibody or rabbit IgG as negative control and decoration with the indicated antibodies (β-actin was used as loading control). Western blot signals were quantified by densitometry and reported in histograms after normalization on anti-TRF2 signals in the IP samples. Bars are SD.

B: PARP1 interaction with TRF1 and TRF2 is not increased by DNA damage. Hela cells were treated with 10 mM HU and Aph for 24 hours, then samples were immunoprecipitated with anti-TRF1 and anti-TRF2 antibodies and decorated with anti PARP1 antibodies. Activation of DDR was checked by wb against γ-H2AX in the input samples. β-actin was shown as loading control. Western blot signals were quantified by densitometry and reported in histograms after normalization on anti-TRF1 and anti-TRF1 signals in the IP samples respectively. Bars are SD.

Supplemental Figure 5

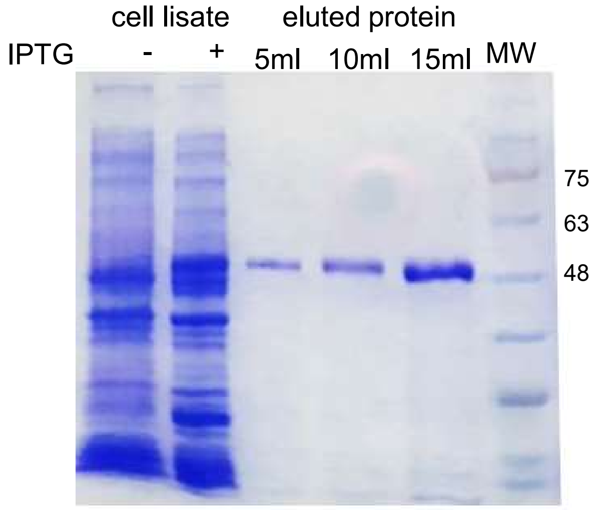

Supplemental Figure 5. Control of purified His-hTRF1. pTrc Hisb vector containing the full length hTRF1 was transformed into Rosetta DE3 competent. Bacterial culture was induced with IPTG, then cells were collected and lysed by sonication. His-tag TRF1 was purified by HisPur™ Ni-NTA Resin and eluted by imidazole. Different quantities of eluted protein were run on PAGE and Coomassie stained to ascertain the purity of protein.

Supplemental Figure 6

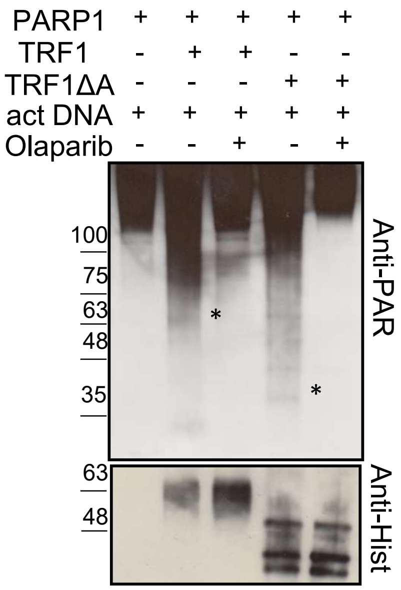

Supplemental Figure 6. TRF1 delta acidic domain is not involved in PARylation. PARP1 high activity purified enzyme was incubated with NAD+ in the PARylation reaction buffer in absence or presence of recombinant His-tag full length or delta acidic TRF1 in presence or absence of activating DNA or Olaparib PARP1 inhibitor. Protein mixtures were resolved on PAGE and decorated with an anti-PAR specific antibody or anti-His antibody to detect TRF1 isoforms where present.

Supplemental Figure 7

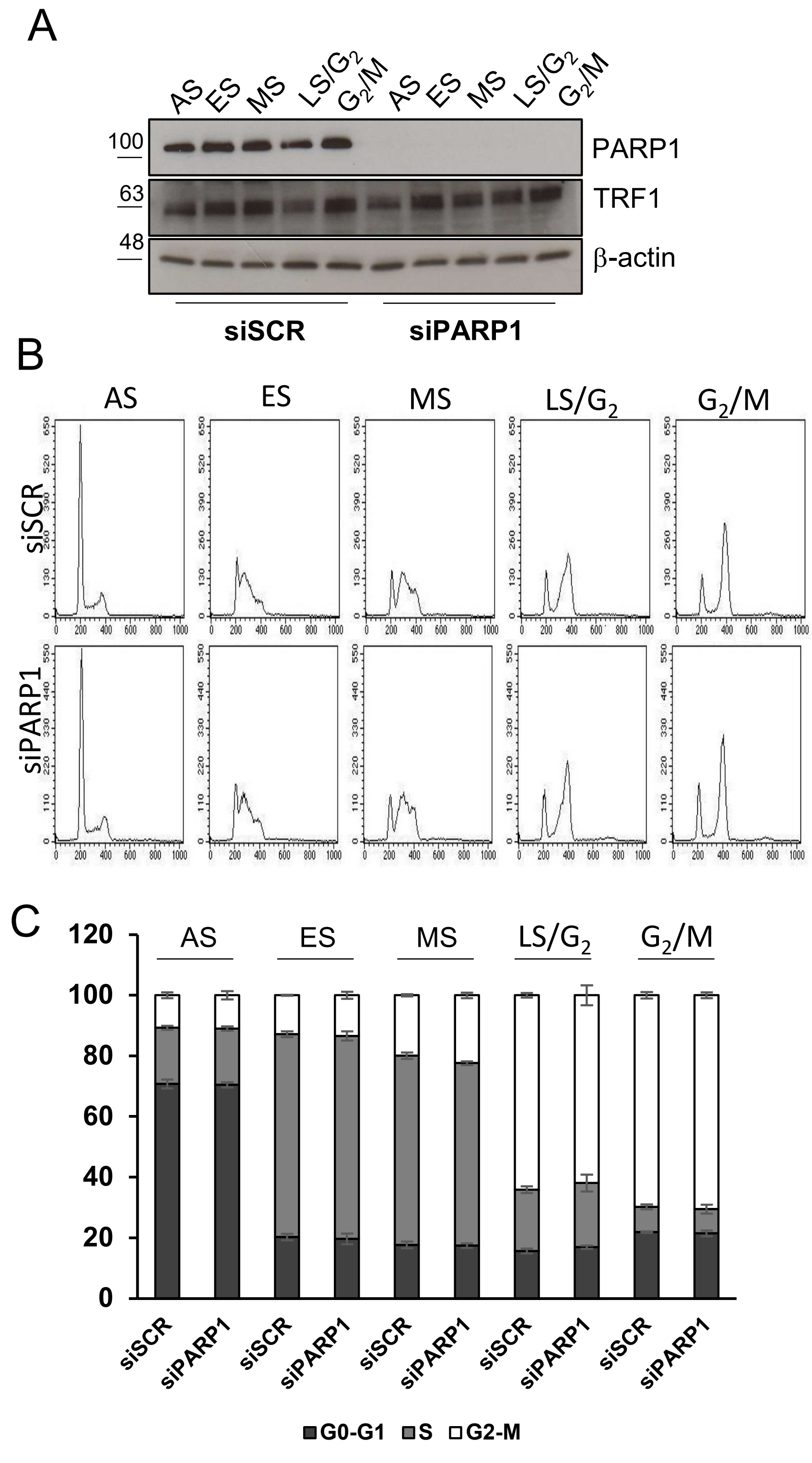

Supplemental Figure 7. HeLa cells were transfected with siSCR and siPARP1 RNA, and synchronized by double thymidine as indicated in Figure 3 A. At each endpoint, the samples were splitted and processed for WB analysis to control PARP1 downregulation (**A,** β-actin was used as loading control), FACS analysis (**B** and **C**). In **B**, the cell cycle progression was measured by PI staining (one representative of three independent experiments is shown), and the fraction of cells in the different cell cycle phases was quantified and reported in **C**, (the mean of three independent experiments is shown, bar are SD.

Supplemental Figure 8

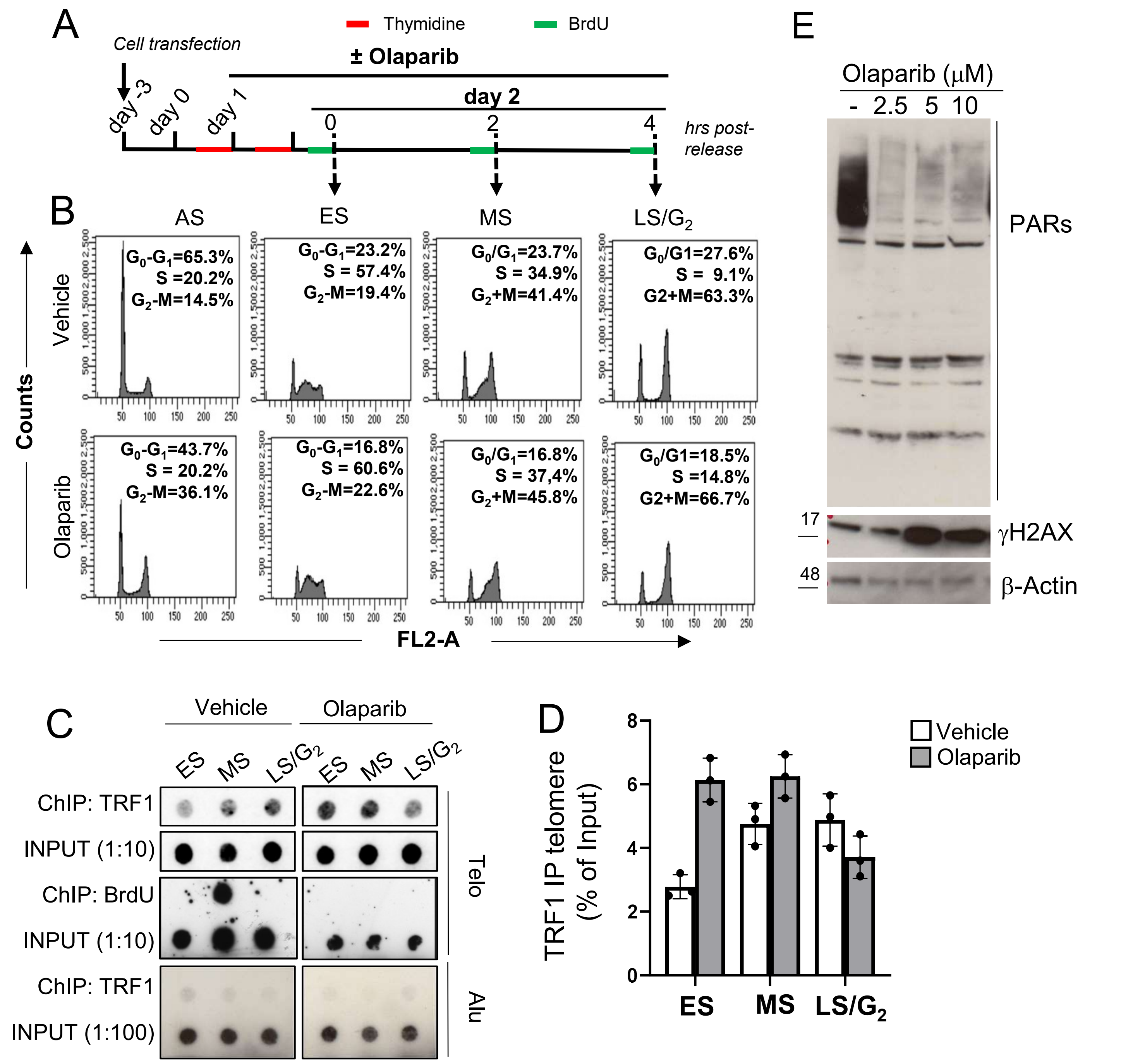

Supplemental Figure 8. Olaparib perturbs telomere replication in absence of massive DNA damage induction. A: Hela cells were synchronized in the early S phase by double thymidine block and then released as indicated. 2 μM Olaparib was administered from the second thymidine block. 1 hr pulse of BrdU incorporation was administered before each endpoint. Samples collected underwent PI staining and flow cytometry to control cell cycle distribution (**B**) or ChIP with an anti-TRF1 specific antibody (**C** and **D**). Immunoprecipitated chromatin samples were dot blotted and processed first by western blot with an anti-BrdU specific antibody, and then hybridized with ^{32}P labelled telo or alu probes (**C**). Signals were quantified by densitometry and the fraction of TRF1 immunoprecipitated telomeric chromatin was reported in graphs as the percentage of each respective input after normalization on the Alu signals (**D**). **E**: Hela cells were treated with the indicated doses of Olaparib. Then cells were collected and processed for western blot analysis against anti-PAR antibody and γ-H2AX. β-actin was used as a loading control.

Supplemental Figure 9

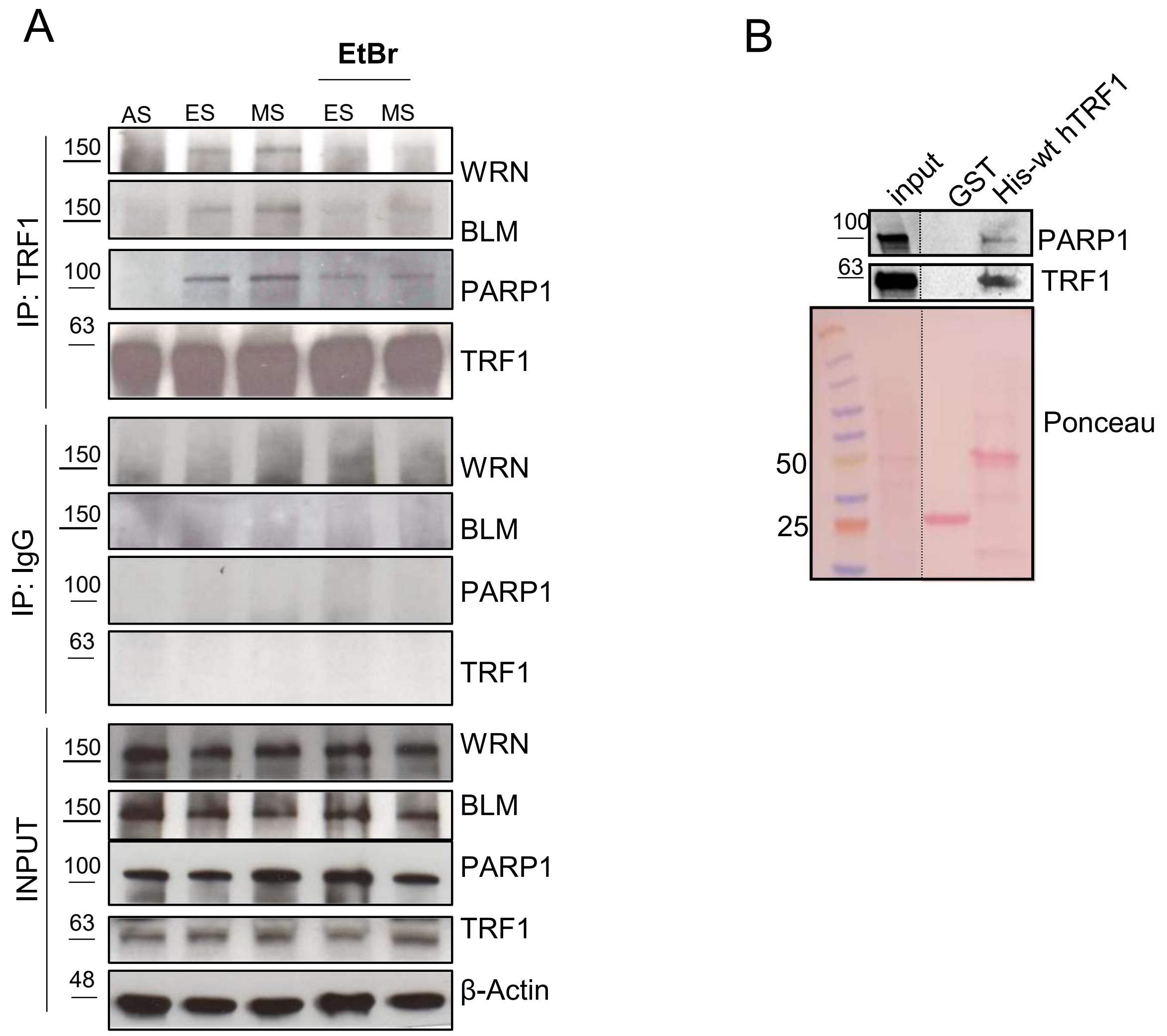

Supplemental Figure 9. PARP1/TRF1 interaction is direct but S-phase dependent stabilization of interaction and helicase recruitment requires DNA. A: Hela cells were synchronized as above described and collected at the indicated endpoints. Cell lysates were immunoprecipitated with anti TRF1 in presence or absence of EtBr, and than IP-ed complex were resolved on gradient acrylamide gel and processed for WB analysis with the indicated antibodies, β-actin was used as a loading control. **B:** Asynchronous Hela cells were lysed in a high salt buffer to disrupt protein-protein and protein-DNA interaction. Then the lysate was diluted in low salt buffer and incubated with recombinant His-tagged wt hTRF1 conjugated with a HisPur™ Ni-NTA Resin or recombinant GST conjugated with glutathione-sepharose beads as negative control. Pulled down proteins were resolved on SDS PAGE and processed for ponceau staining to evidence GST and TRF1 quantities in the sample and for wb analysis with the indicated antibodies.

Supplemental Figure 10

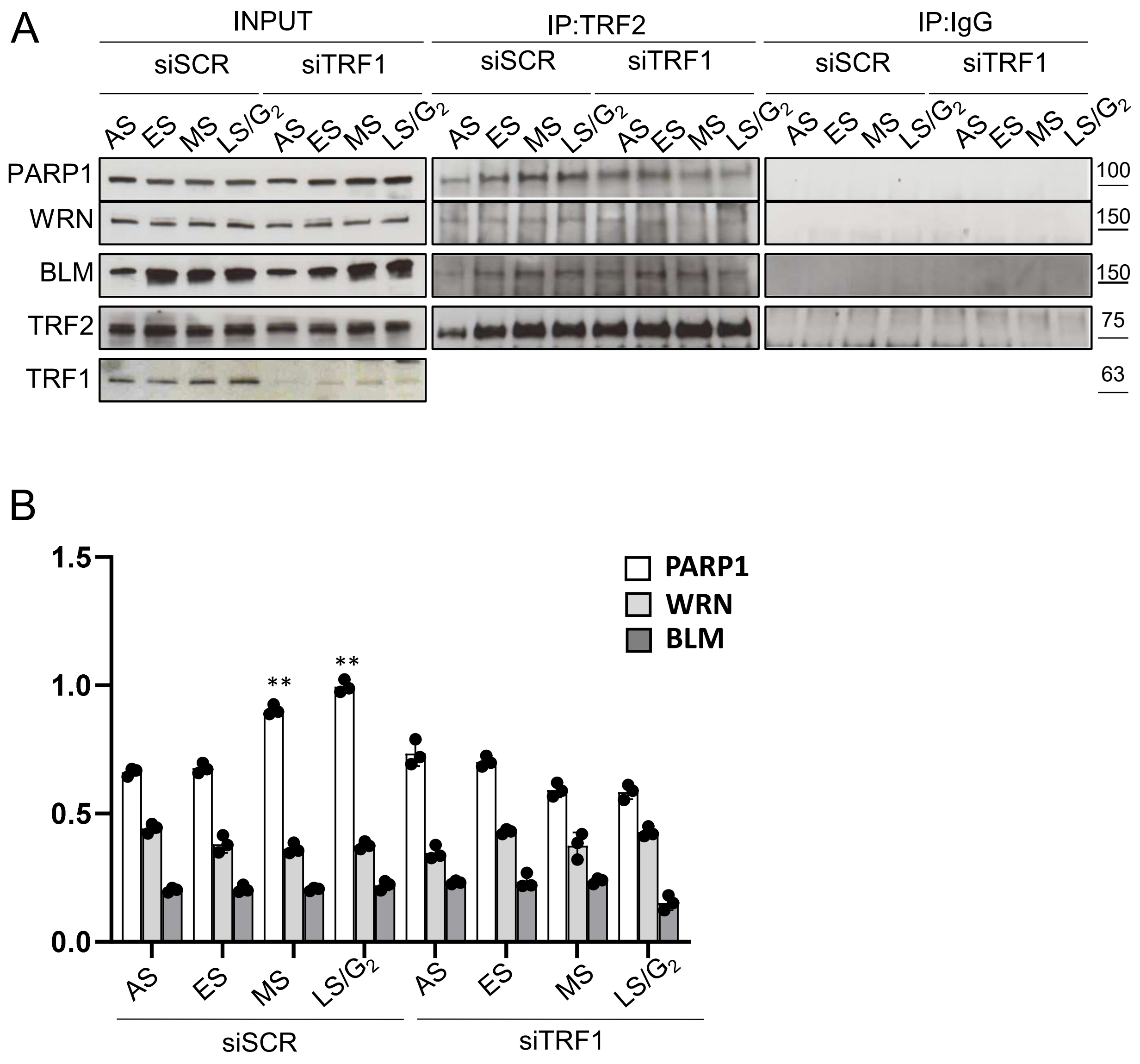

Supplemental Figure 10. TRF2 interaction with PARP1, WRN and BLM is not affected by cell cycle progression or TRF1 interference. A: Hela cells were interfered for TRF1 or a SCR sequence as control. Then cells were synchronized in the early S phase by double thymidine block and released as above described. Samples collected at the indicated endpoints underwent IP with an anti-TRF2 specific antibody followed by WB analysis against the indicated proteins. **B**: Western blot signals were quantified by densitometry and reported in histograms after normalization on anti-TRF2 signals in the IP samples. Bars are DS. **P<0,001. Unpaired T-test

Supplemental Figure 11

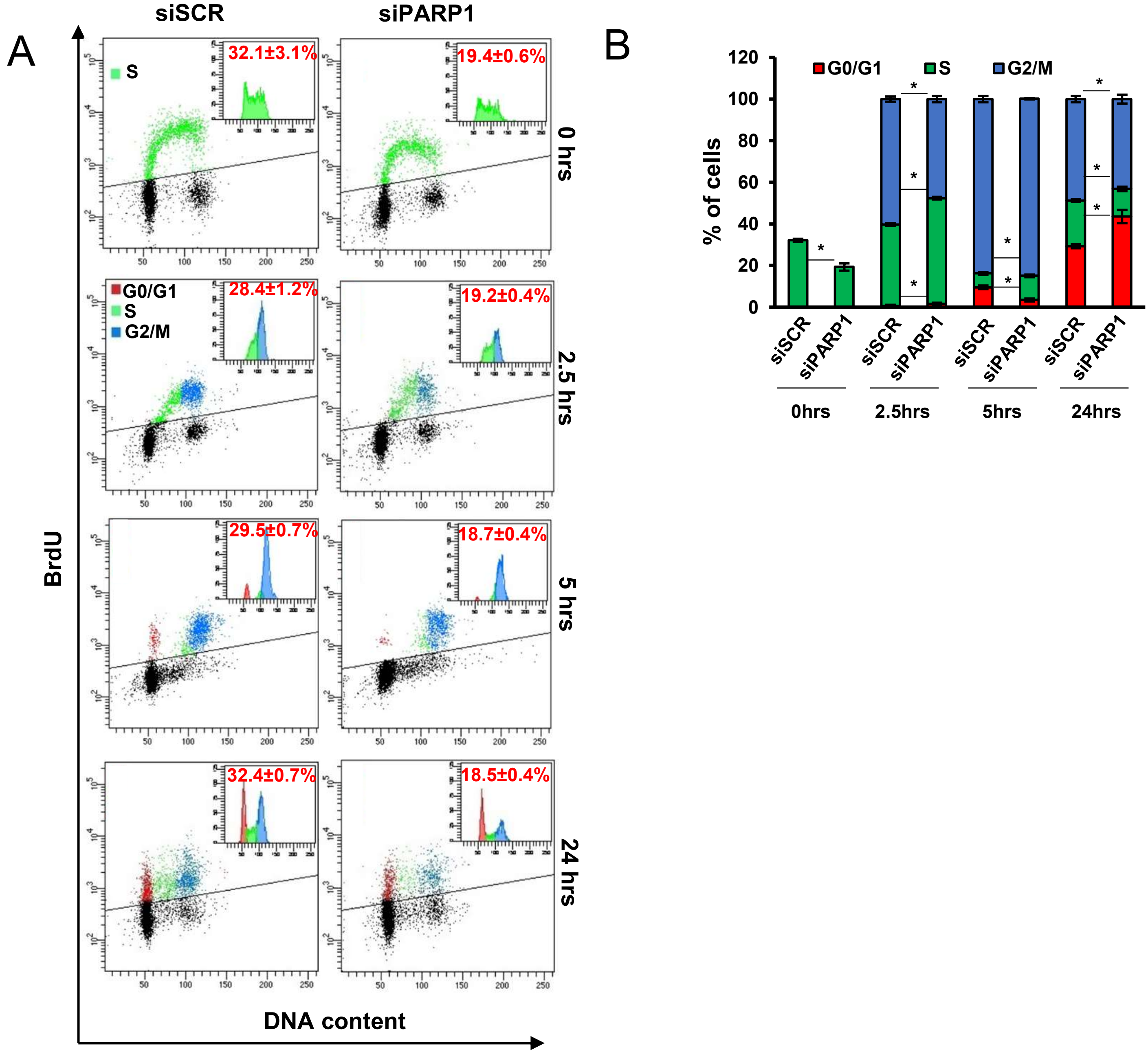

Supplemental Figure 11. PARP1 depletion induces a slight delay of cell cycle progression. A: HeLa cells were transfected with siRNAs against siPARP1 and a scrambled sequence, then cells were pulsed with BrdU for 15 min, and, after the indicated intervals in BrdU-free medium, the DNA was denatured, incubated with anti-BrdU antibody and PI stained. BrdU− (black area) and BrdU+ (multicolor area) populations were separated by analytical sorter in bi-parametric distribution. Graph inserts on top-right show the DNA content of BrdU-positive cells, and the cell cycle phases distribution. Flow cytometry data analysis is built upon the principle of gating and the percentages of G0-G1 (red) S (green) and G2/M (blue). The percentages of cell cycle phases distribution at each endpoint are reported in histograms (B). One representative of three independent experiments with similar results is shown. Bars are SD. P value was determined by unpaired two tailed t-student test *P≤0.05.

Supplemental Figure 12

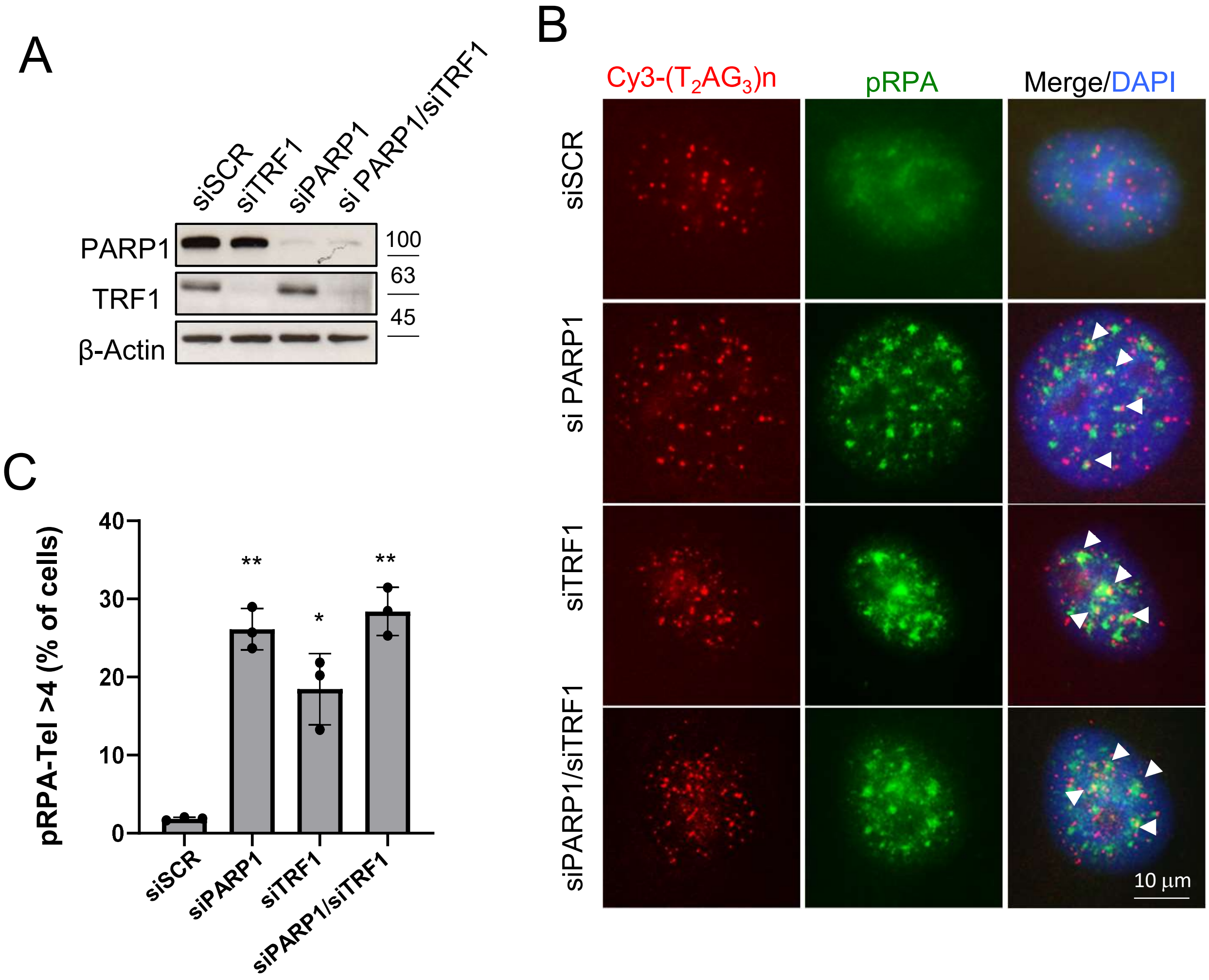

Supplemental Figure 12. PARP1 depletion induces a replication dependent DNA damage. HeLa cells were transfected with siRNAs against siPARP1 and siTRF1 alone and in combination and against a scrambled sequence. Efficiency of knock-down by RNA interference was controlled by WB analysis (**A**, β-actin was used as loading control). After 48 hours post transfection, parallel samples were fixed and processed for IF-FISH against pRPA and telomere repeats by a Cy3-Telo PNA probe and counterstained with DAPI. Signals were acquired by Leica Deconvolution fluorescence microscope at 63X magnification, representative images are shown in panel **B**, arrows indicate co-localizations. The percentage of cells displaying >4 pRPA/telomere co-localizations was scored and reported in histograms in **C**. The average of three independent experiments is shown, bars are SD. P value was determined by unpaired two tailed t-student test $^*P \leq 0.05$, $^{**} P \leq 0.01$.

Supplemental Figure 13

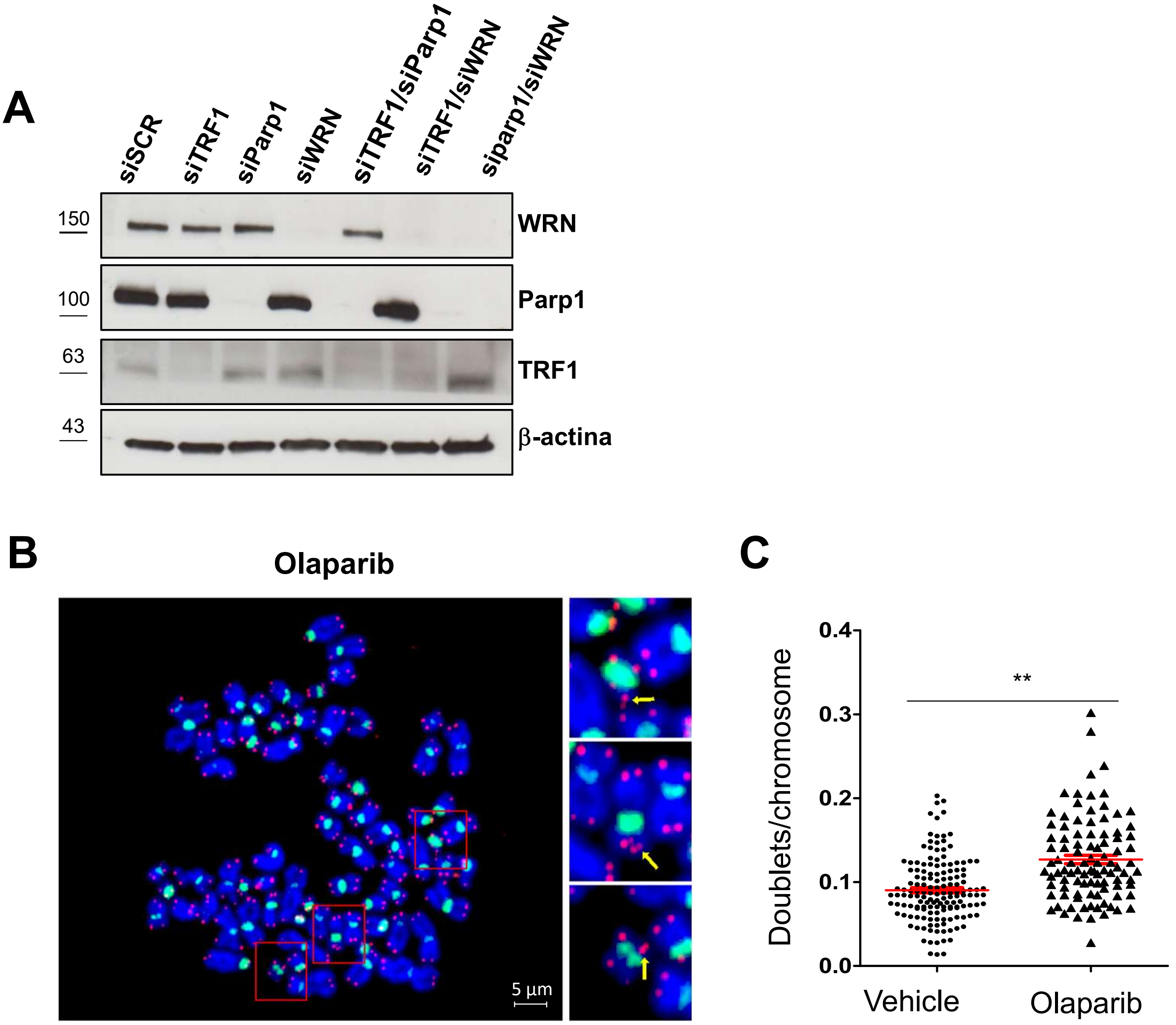

Supplemental Figure 13. A. Interference efficiency of metaphase samples. Samples of cells treated as in Figure 6 were checked for interference efficiency by WB analysis.

B and C. Olaparib induces telomere fragility phenotype. HeLa cells were treated with 2 µM Olaparib for 24 hours and then metaphases were collected and processed for FISH for pantelomeric/pancentromeric staining and counterstained with DAPI. Representative images at 100X magnification are shown in **B**. **C**: Telomere doublets were scored and reported in graphs as the number of doublets/chromosomes. Two pulled independent experiments were plotted, red bars are means, P value was determined by unpaired two tailed t-student test. ** $P \leq 0.01$.

Supplemental Figure 14

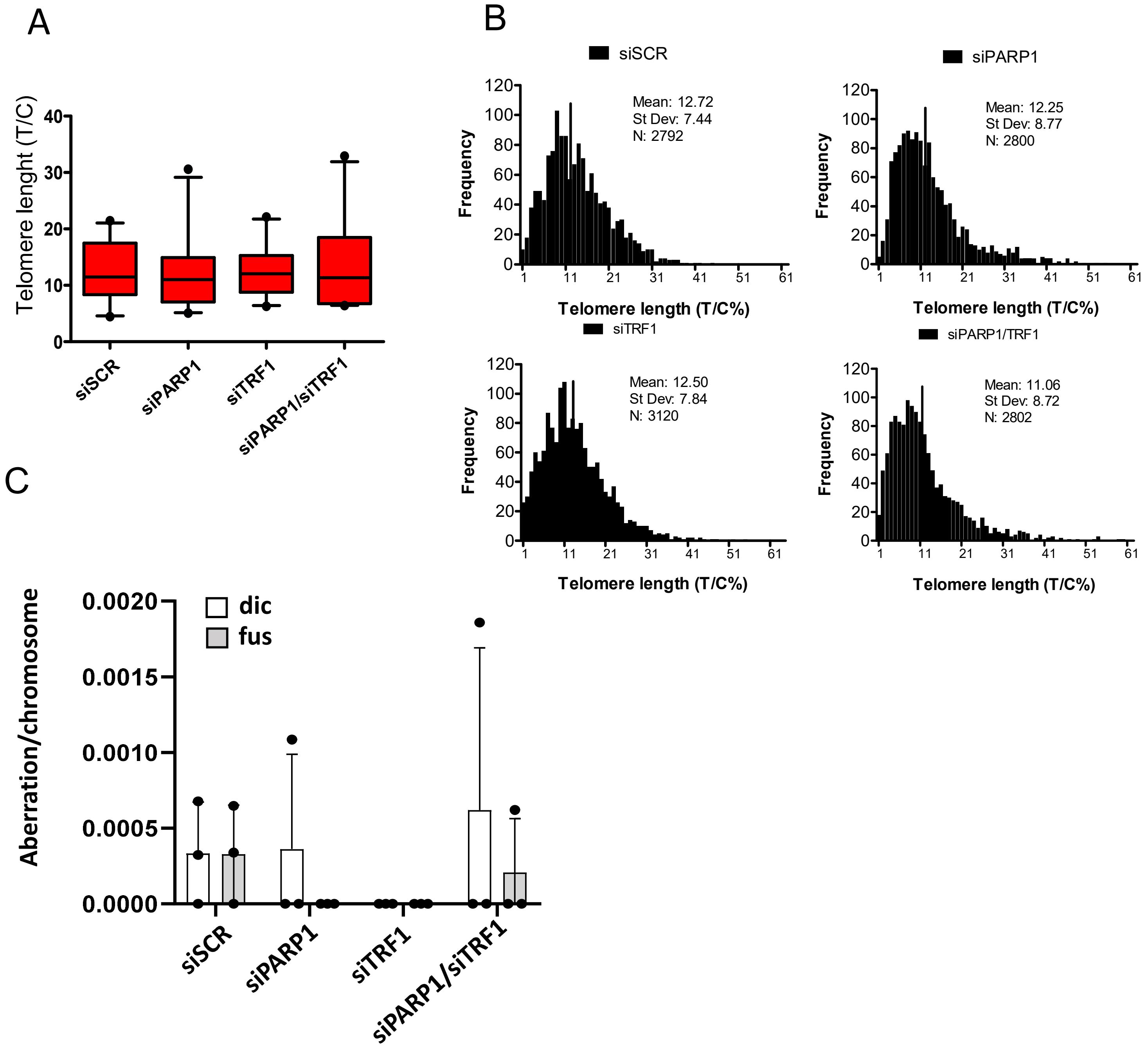

Supplemental Figure 14. Effect of TRF1/PARP1 interference on telomere length and other telomere aberrations. Hela cells interfered for 72 hours with the indicated siRNAs were synchronized in metaphase and processed for FISH analysis with pan-centromeric and pan-telomeric probes. Images of metaphases were acquired and analyzed for telomere length (**A** and **B**) and scored for the presence of dicentric chormosomes (dic) and telomeric fusions (fus)(**C**). As shown by the histograms, both telomere length and the number of aberrations/chromosome were not affected by transfection. (test t student P> 0,1)

Supplemental Figure 15

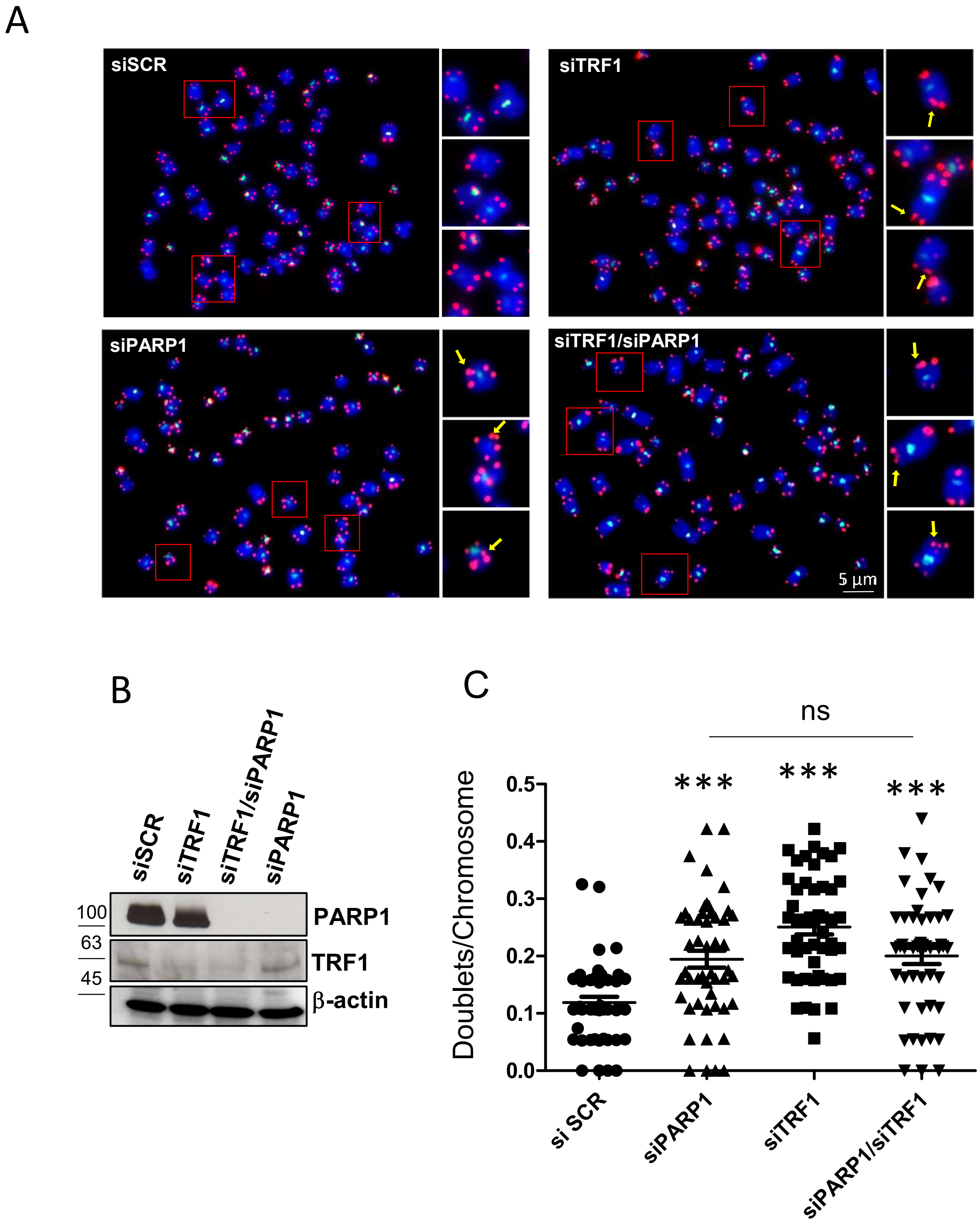

Supplemental Figure 15. Telomere fragility induced by TRF1/PARP1 inteference is exacerbated in cells with long telomeres . HeLa 1.3 cells, possessing long telomeres, were transfected as indicated and after 72 hours metaphases were collected and processed for FISH for pantelomeric/pancentromeric staining and counterstained with DAPI. Representative images at 100X magnification are shown in **A**. **B**: A sample of Hela cells treated as in A was processed for WB analysis to control interference efficiency **C**: Telomere doublets were scored and reported in graphs as the number of doublets/chromosomes. 50 metaphases per sample were analyzed. **D:** the graph compares mean and SD of results shown in Figure 7 B and Supplemental Figure 13 C, displaying the difference in the number of doublets/chromosomes between Hela and Hela 1.3 cells. P value was determined by unpaired two tailed t-student test. ** P ≤ 0.001; *** P ≤ 0.0001.

Supplementary Figure 16

Unedited/uncropped western blot gels

Figure 1 e

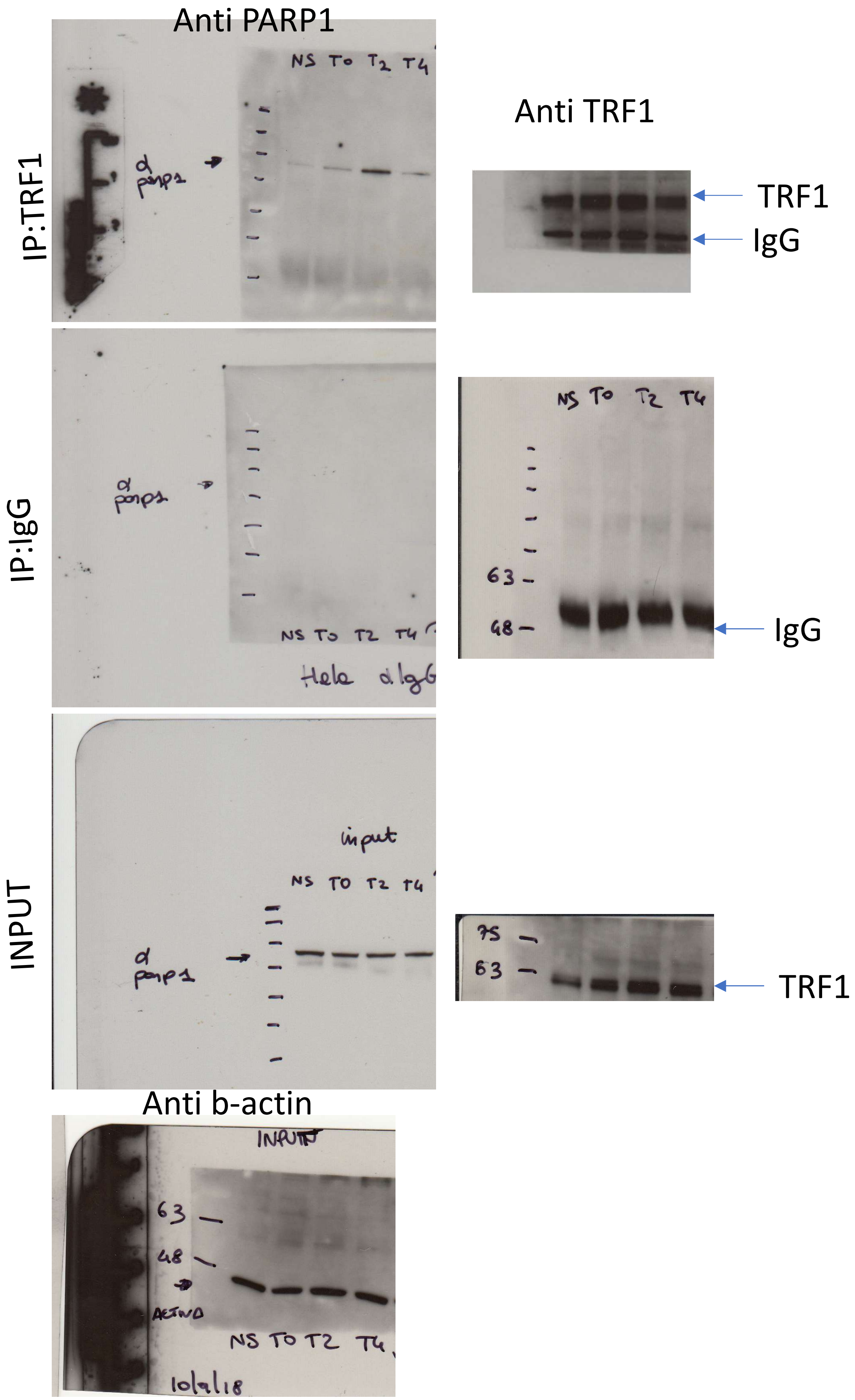
Anti PARP1
Anti TRF1
IP:TRF1
NS T0 T2 T4
TRF1
IgG
IP:IgG
NS T0 T2 T4
63
48
IgG
INPUT
input
NS T0 T2 T4
75
63
TRF1
Anti b-actin
63
48
NS T0 T2 T4

Figure 2 a, b and c

FIG 2 A

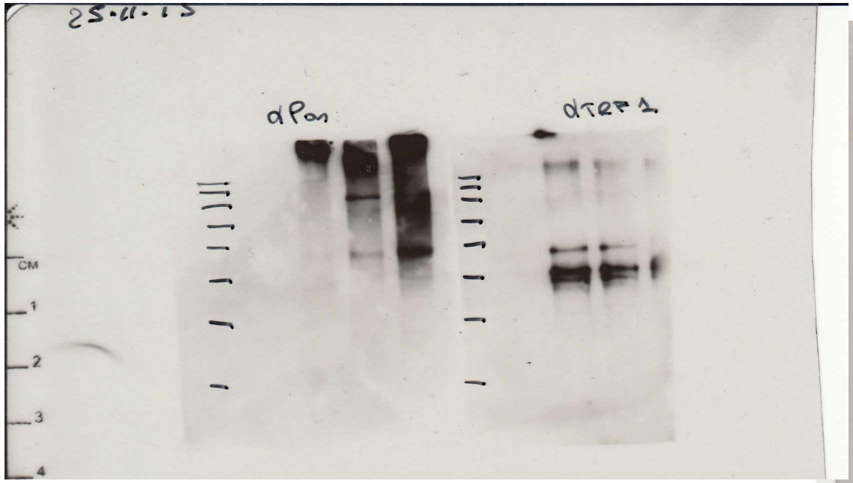

FIG 2 B

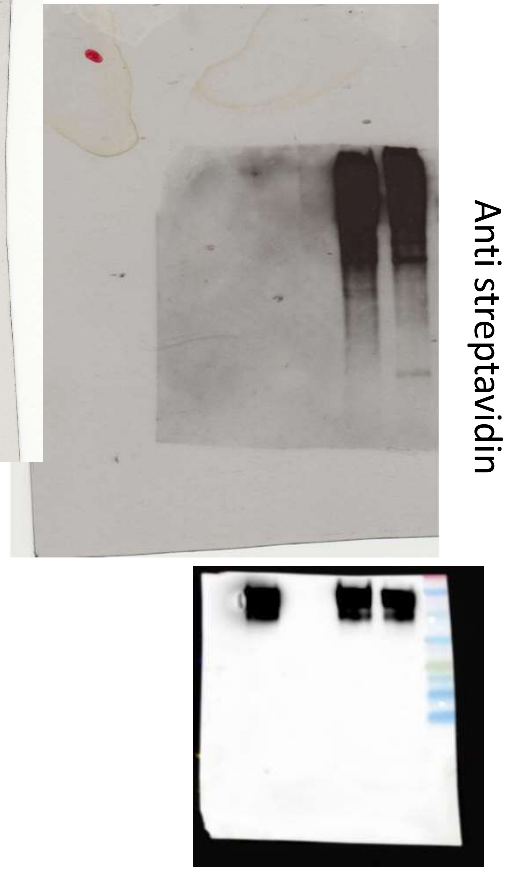

FIG 2 C

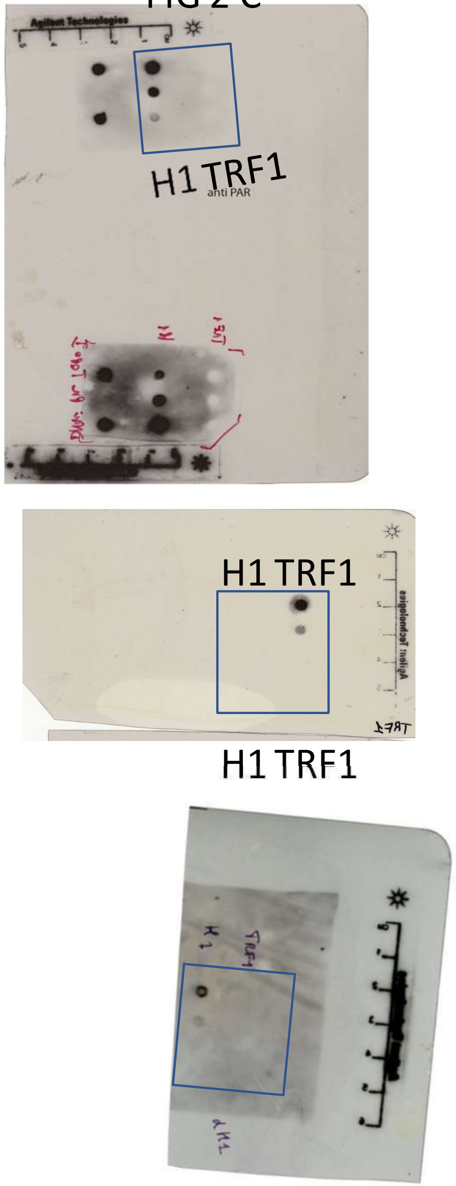

Fig 2 E raw data

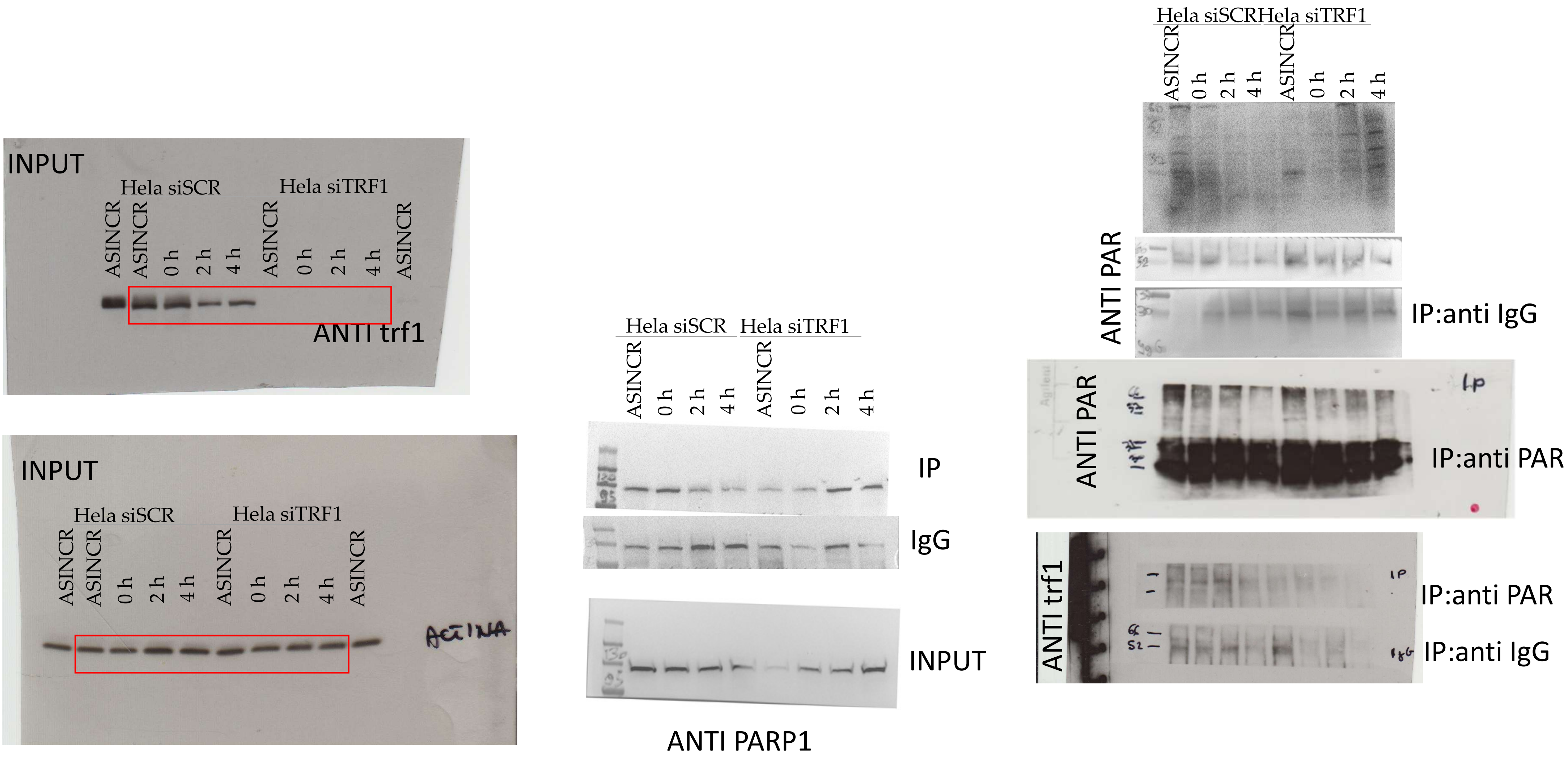

Figure 2 E

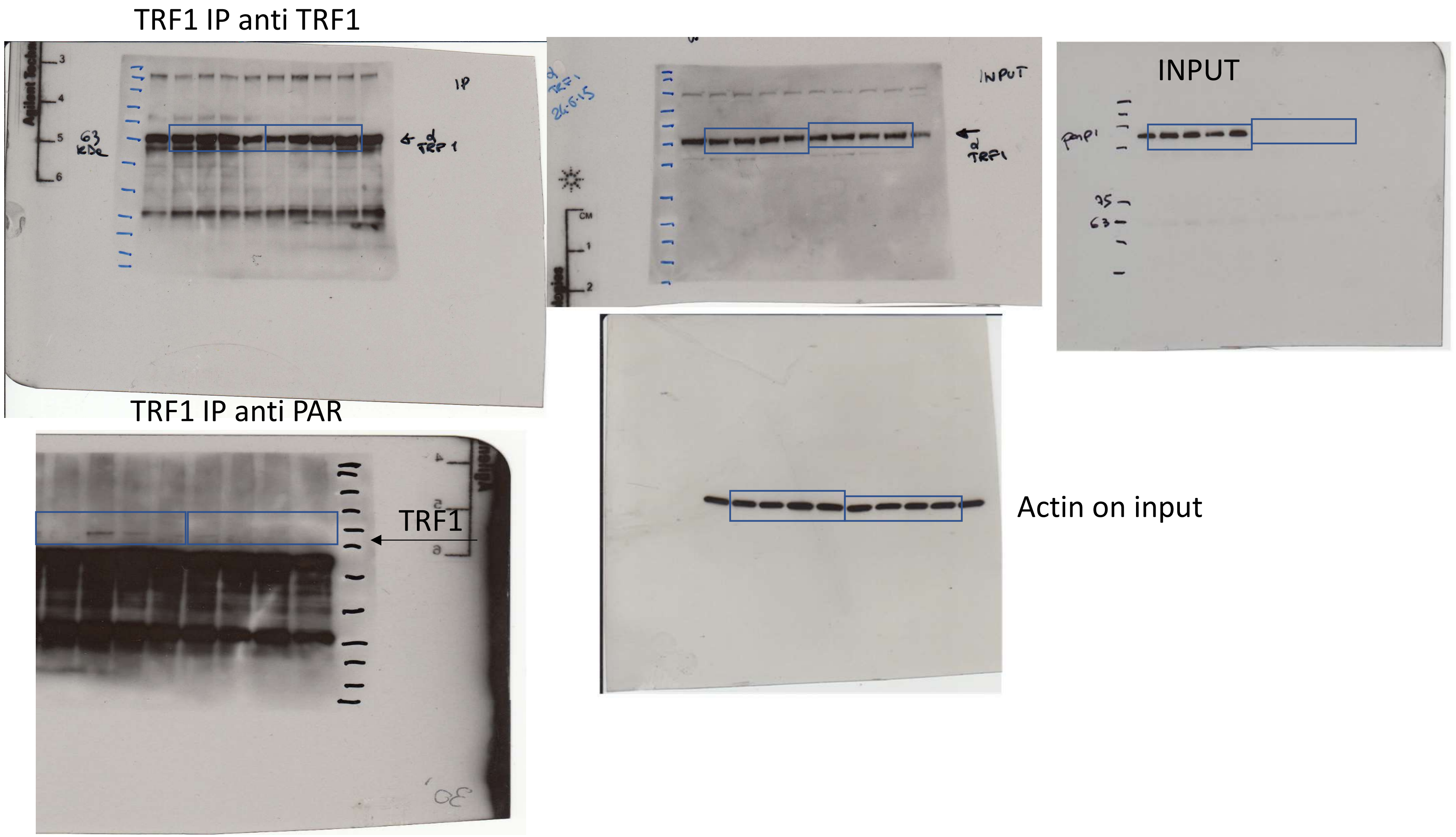
TRF1 IP anti TRF1
INPUT
INPUT
TRF1 IP anti PAR
TRF1
Actin on input

Figure 3 d

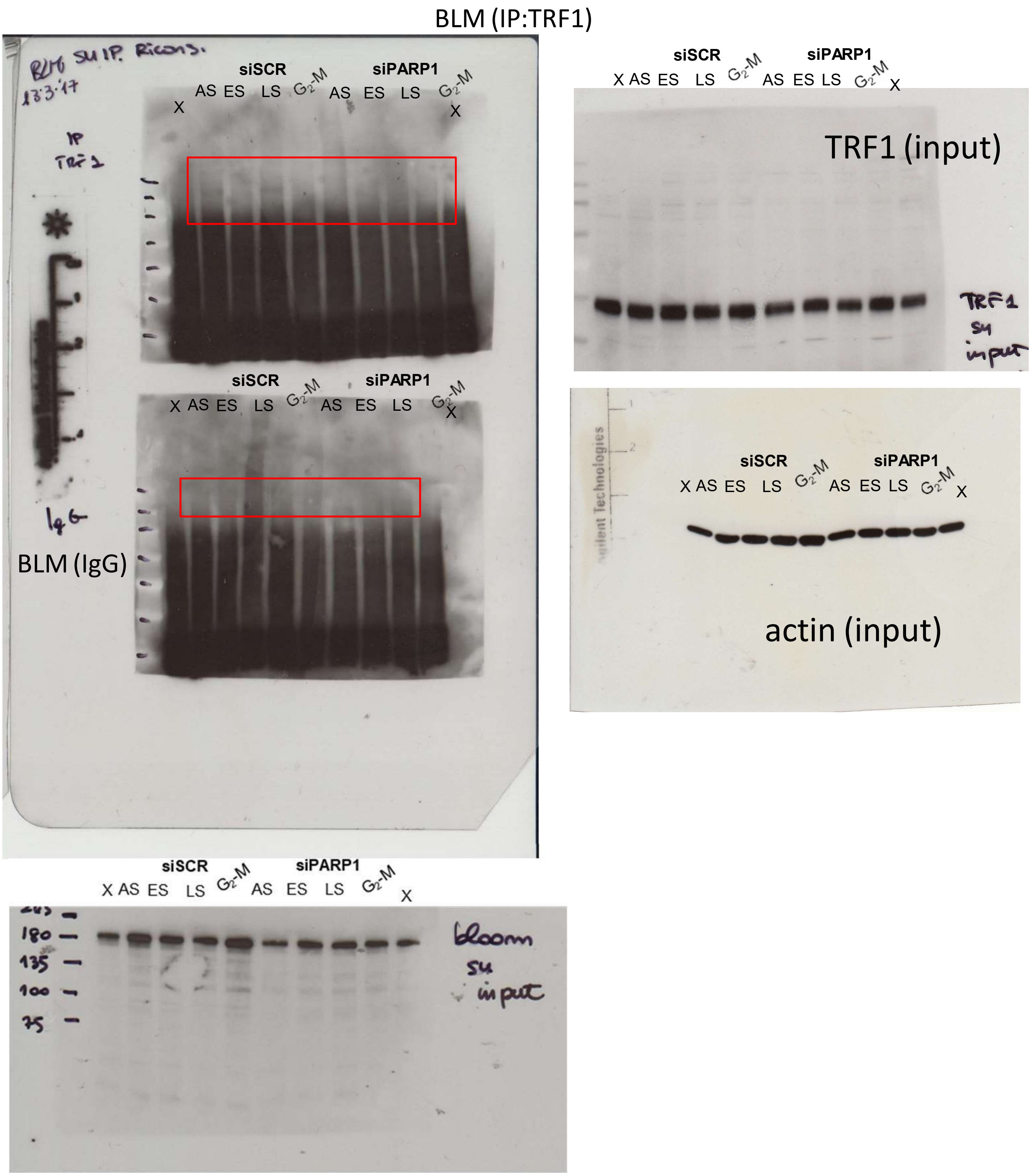
BLM (IP:TRF1)
siSCR
siPARP1
X AS ES LS G2-M AS ES LS G2-M X
IP TRF1
IgG
BLM (IgG)
TRF1 (input)
TRF1 SU input
actin (input)
180
135
100
75
bloom SU input

Figure 3 d

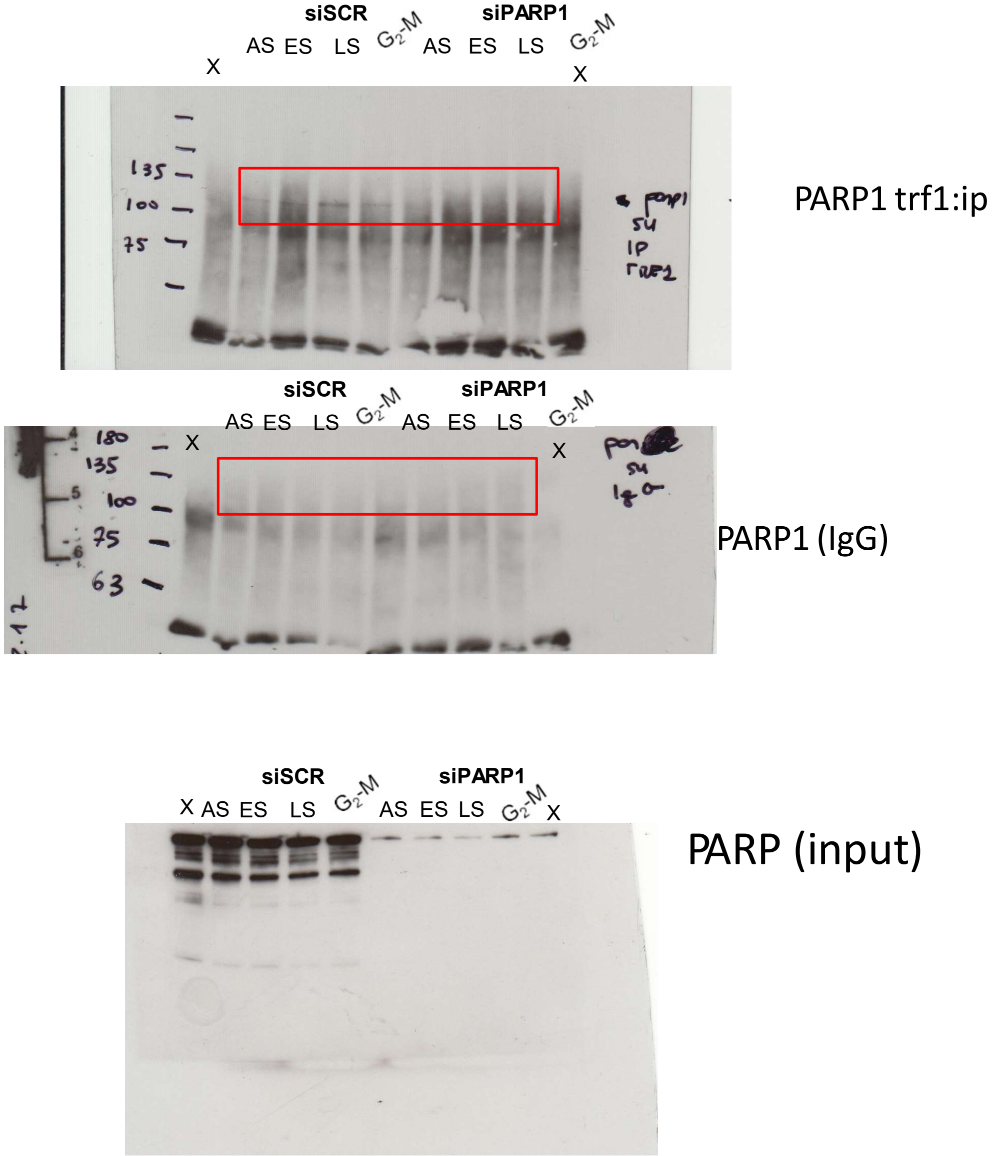
siSCR
siPARP1
X AS ES LS G2-M AS ES LS G2-M X
135
100
75
PARP1 trf1:ip
siSCR
siPARP1
AS ES LS G2-M AS ES LS G2-M
X
X
180
135
100
75
63
PARP1 (IgG)
siSCR
siPARP1
X AS ES LS G2-M AS ES LS G2-M X
PARP (input)

Figure 3 d

WRN (IP:TRF1)

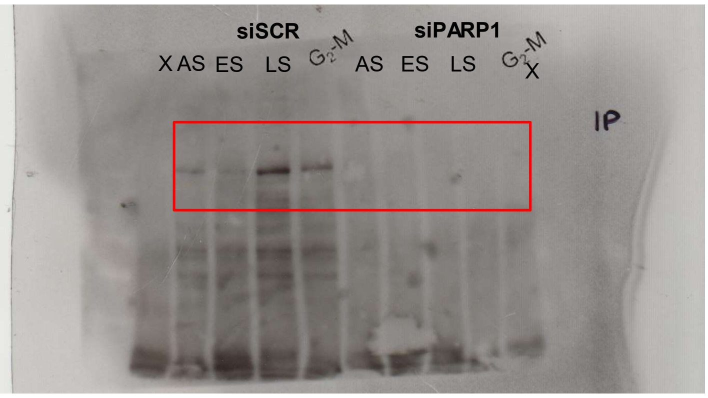

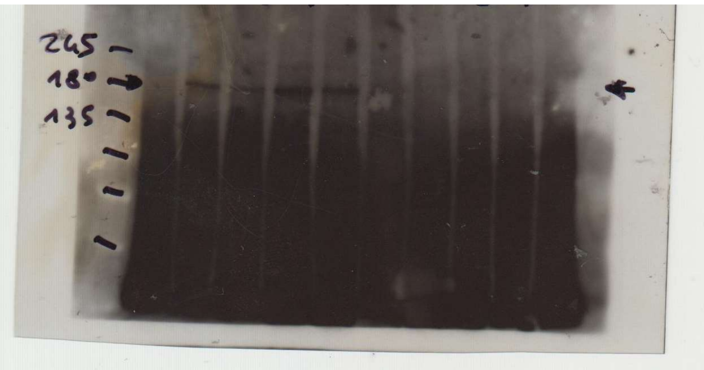

WRN (IgG)

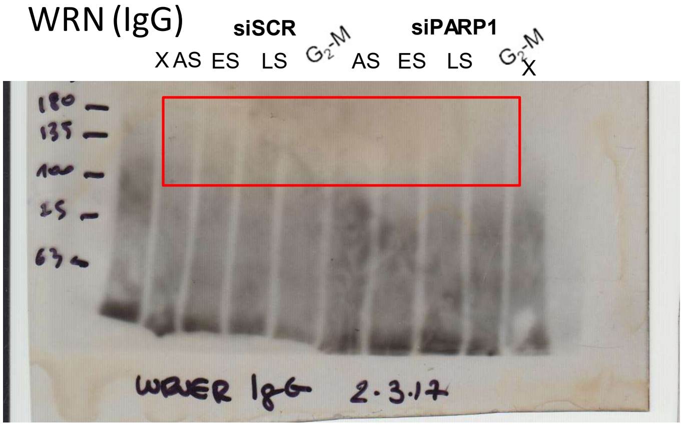

WRN INPUT

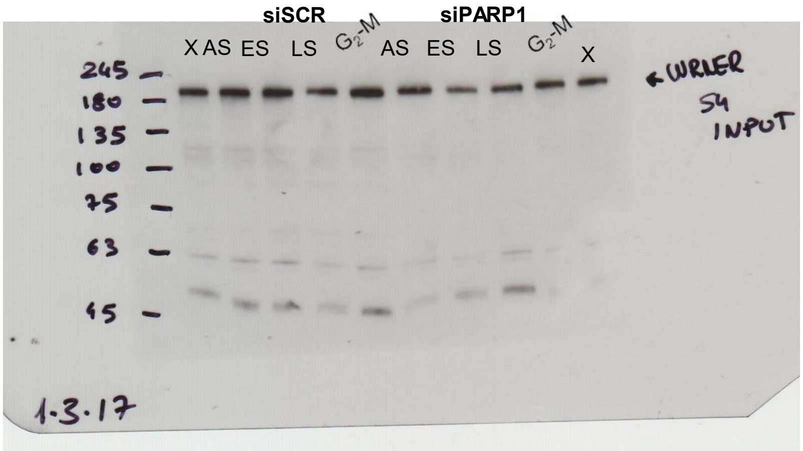

Figure 3 e

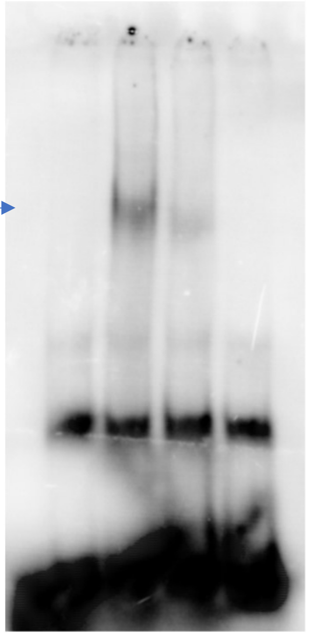

Oligo DNA

Figure 5 b

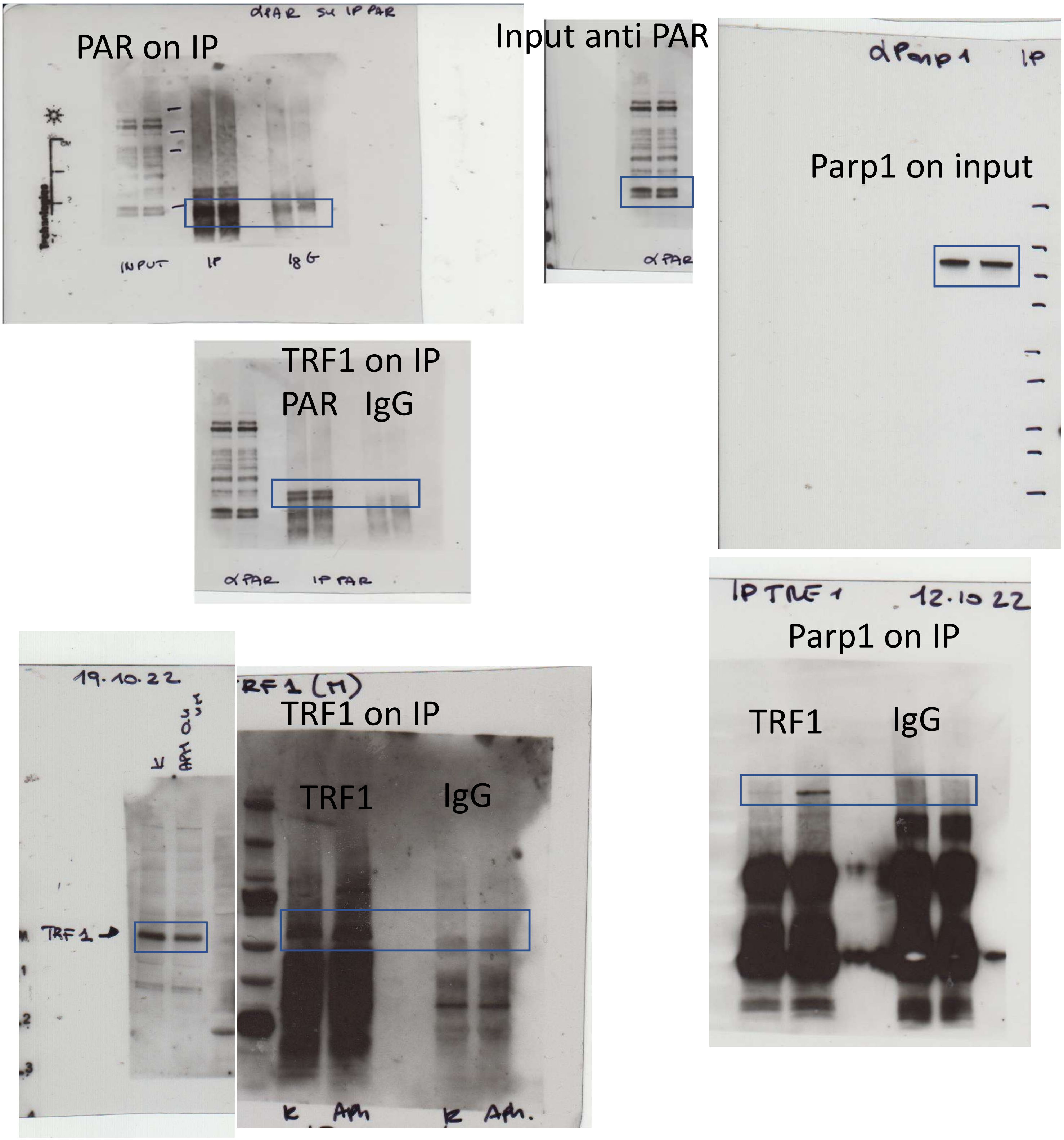
PAR on IP
INPUT
IP
IgG
Input anti PAR
αPAR
αParp1
IP
Parp1 on input
TRF1 on IP
PAR
IgG
αPAR
IP PAR
IP TRF1
12.10.22
Parp1 on IP
TRF1
IgG
19.10.22
TRF1
TRF1 on IP
TRF1
IgG
K
APh
K
APh.

Supplemental figure 2

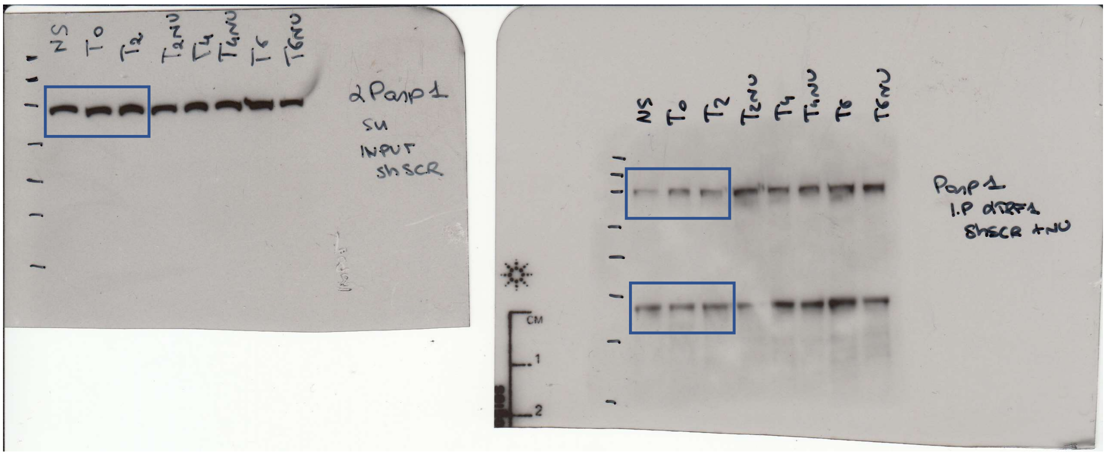

Supplemental figure 4a

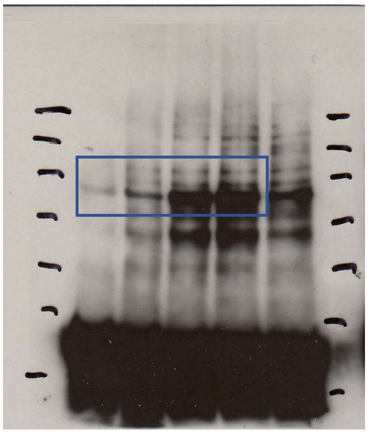

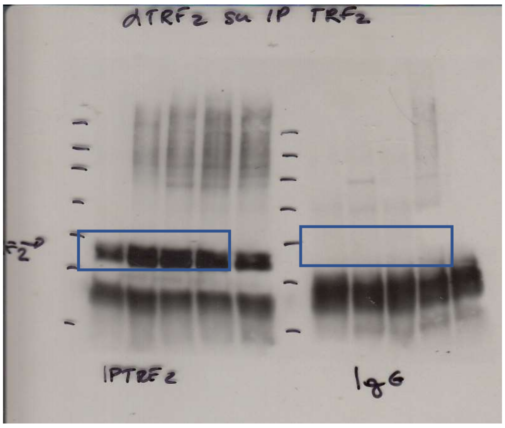

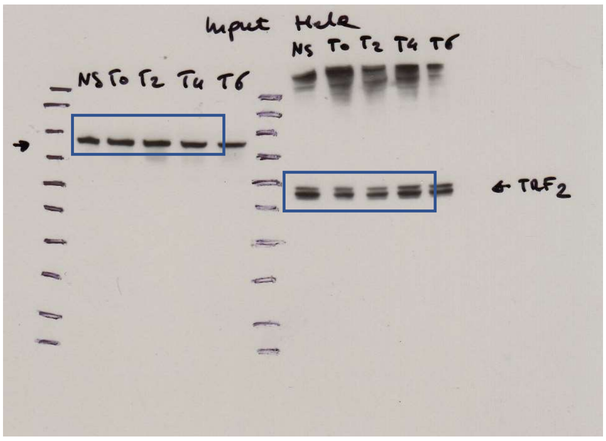

Supplemental figure 4b

parp1

IP:TRF1

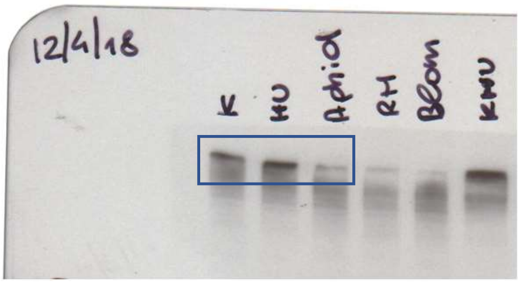

TRF1

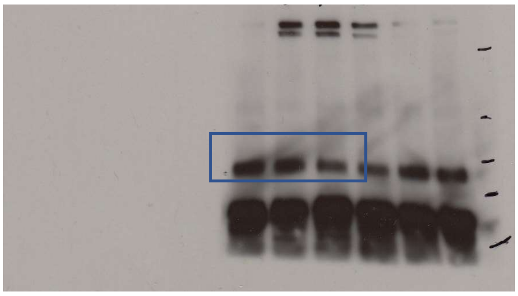

IP:IgG

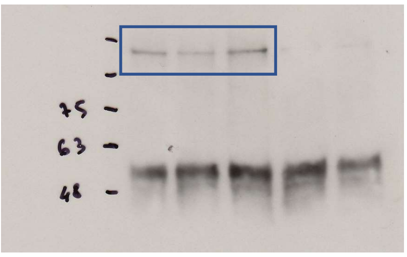

TRF2

IP:TRF2

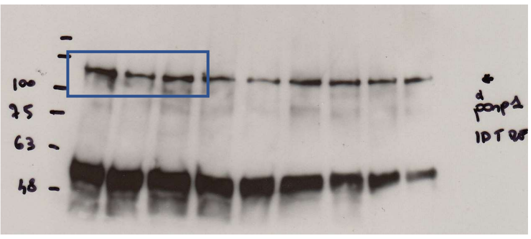

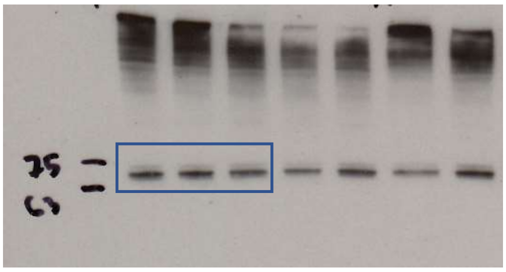

γH2AX

INPUT

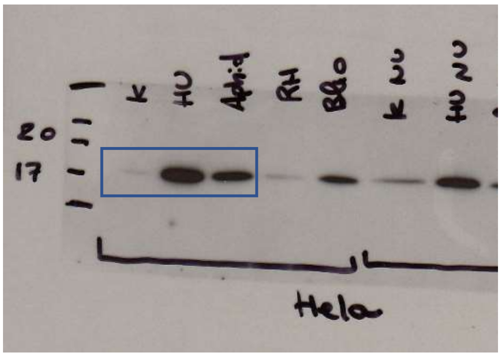

Supplemental figure 6

Anti PAR

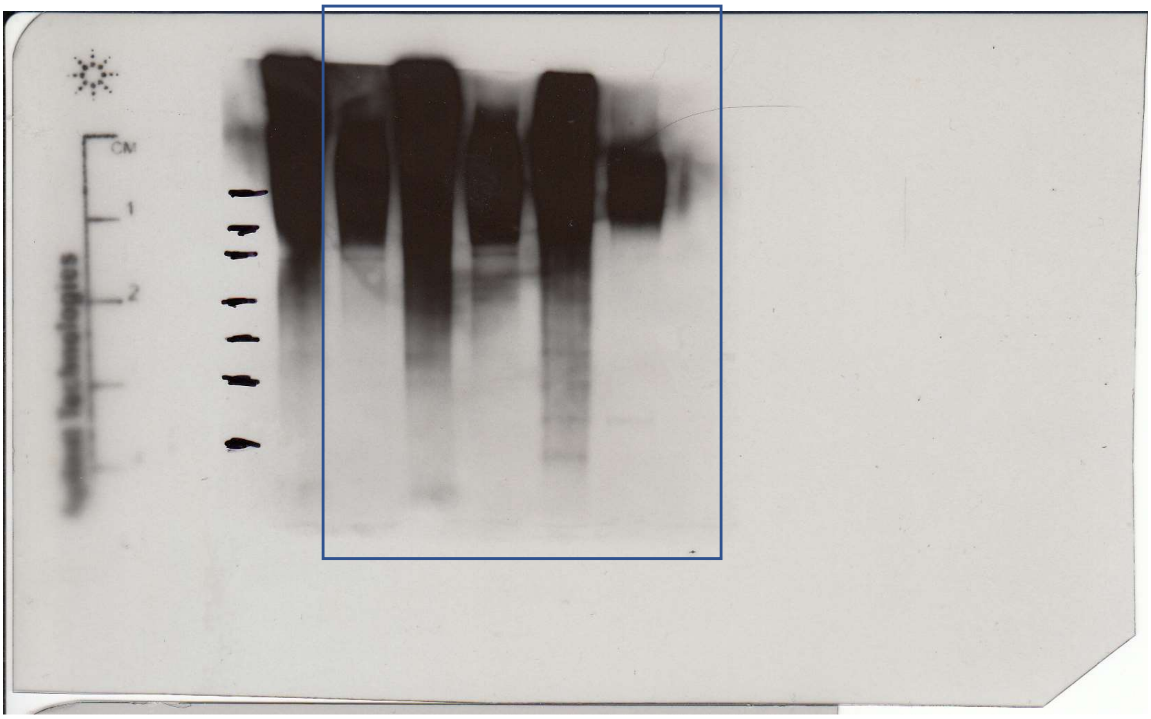

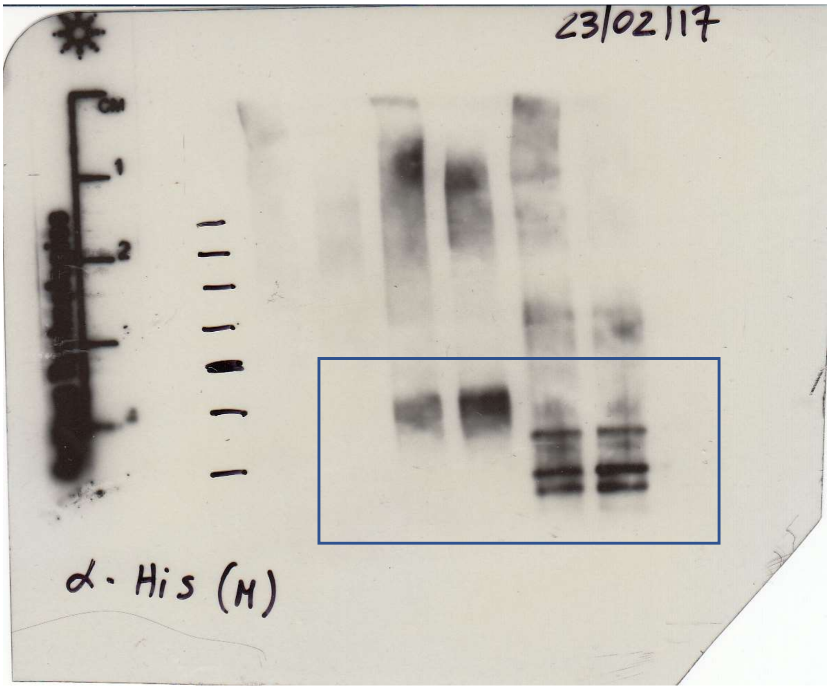

Supplemental figure 7

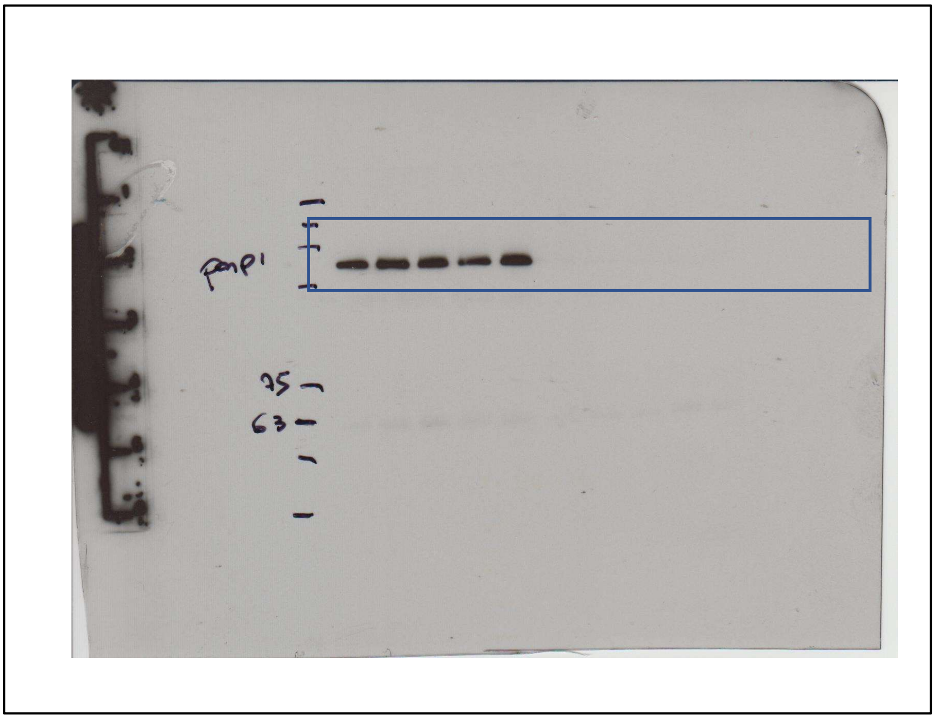

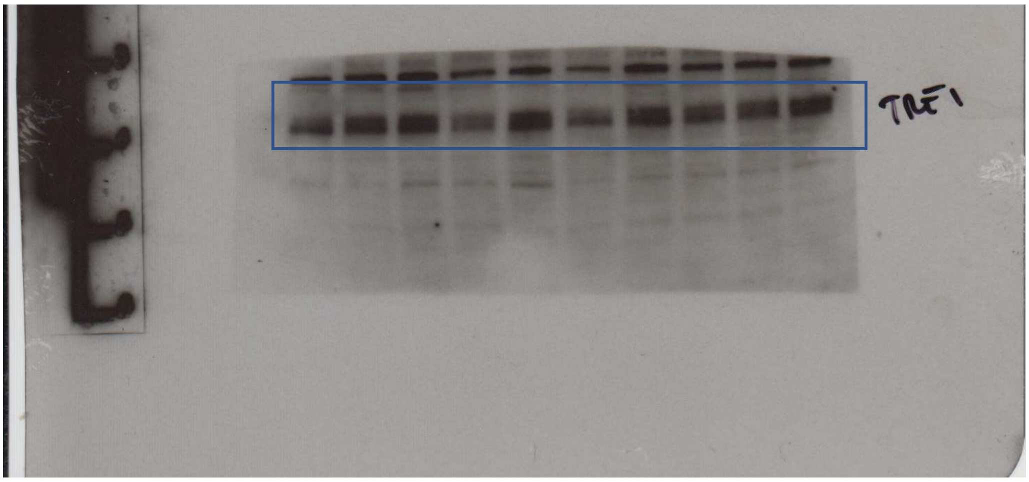

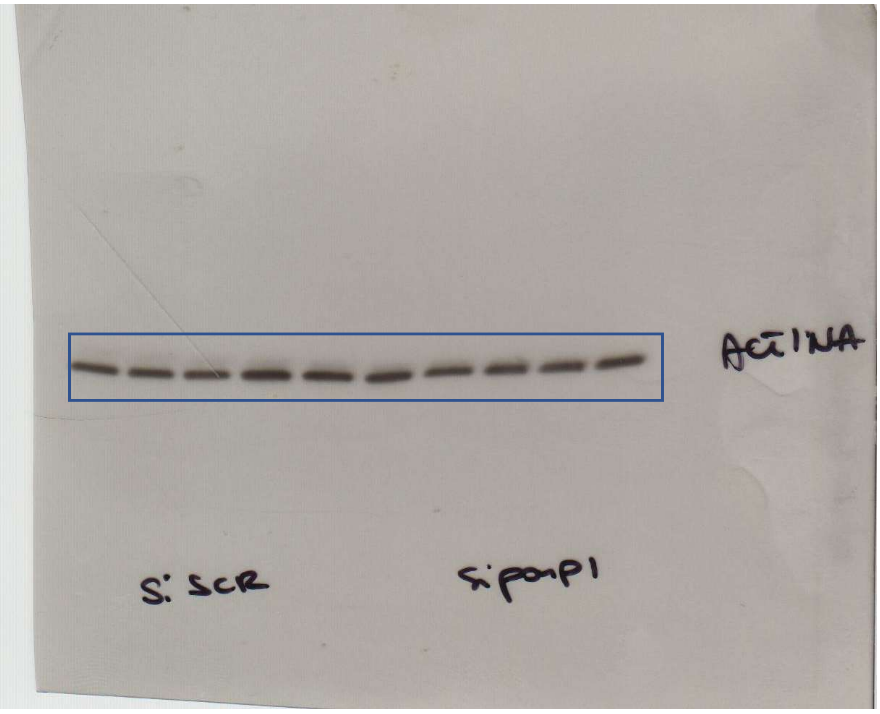

Supplemental figure 8

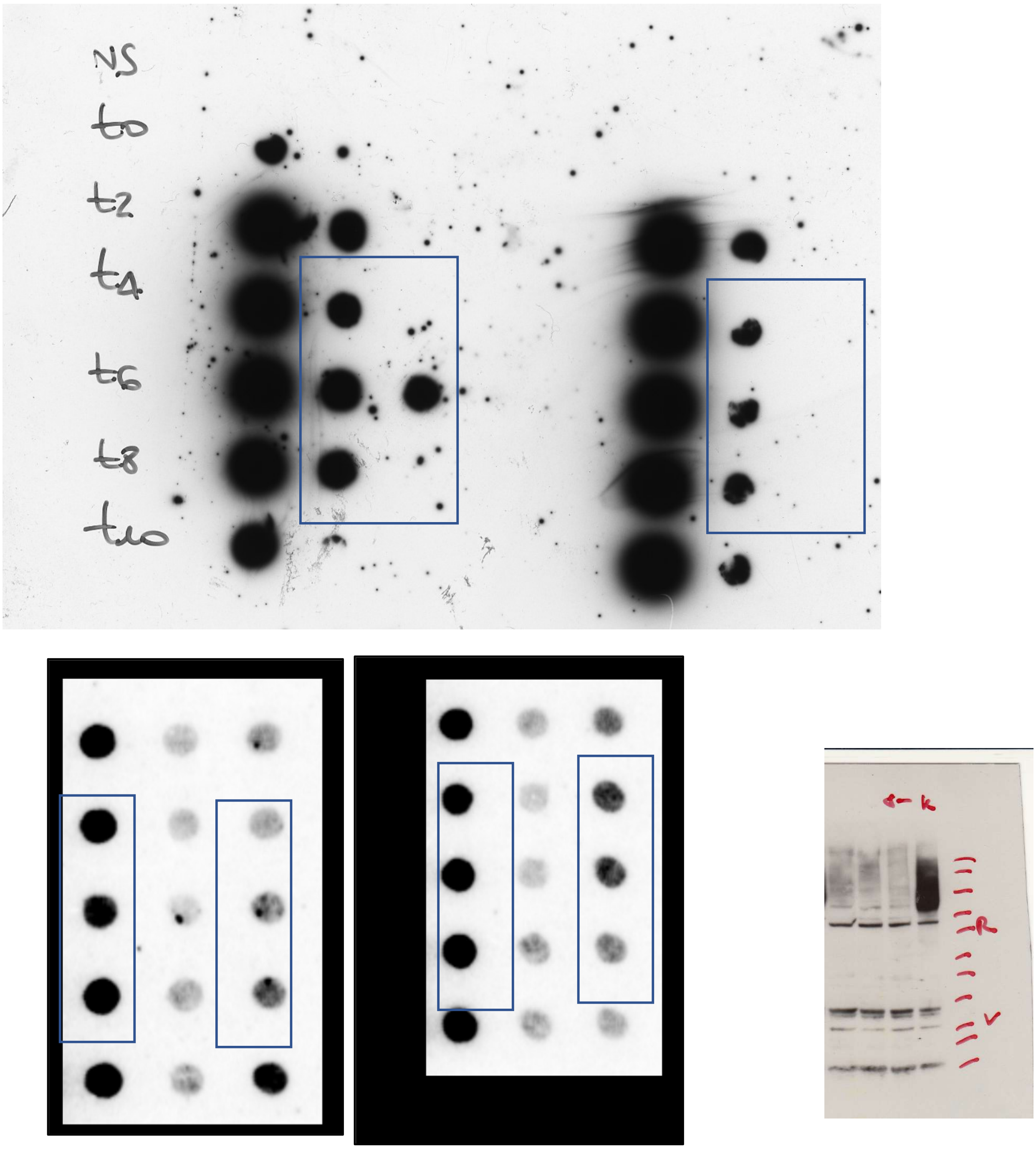

Supplemental figure 9

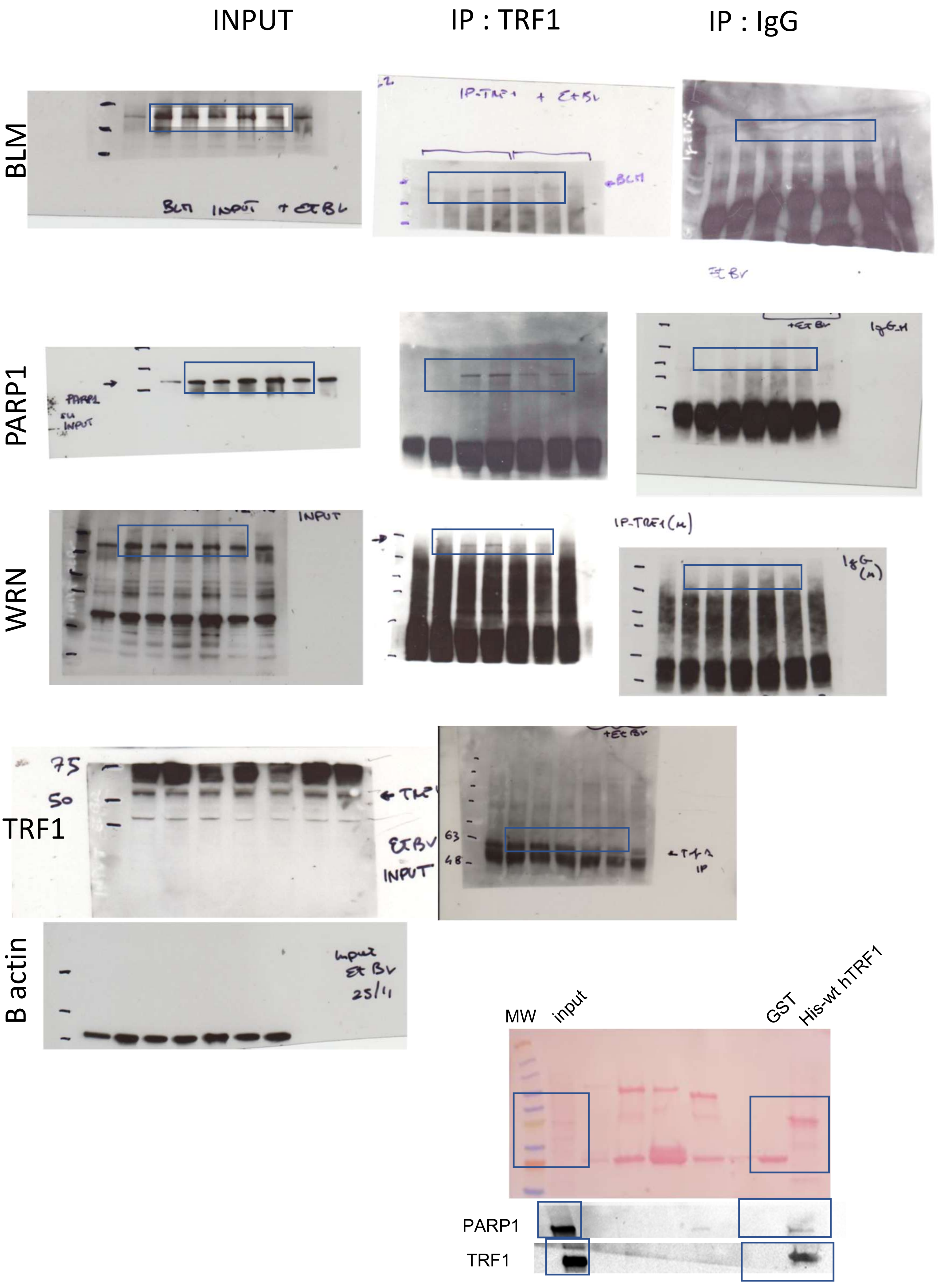

Supplemental figure 10

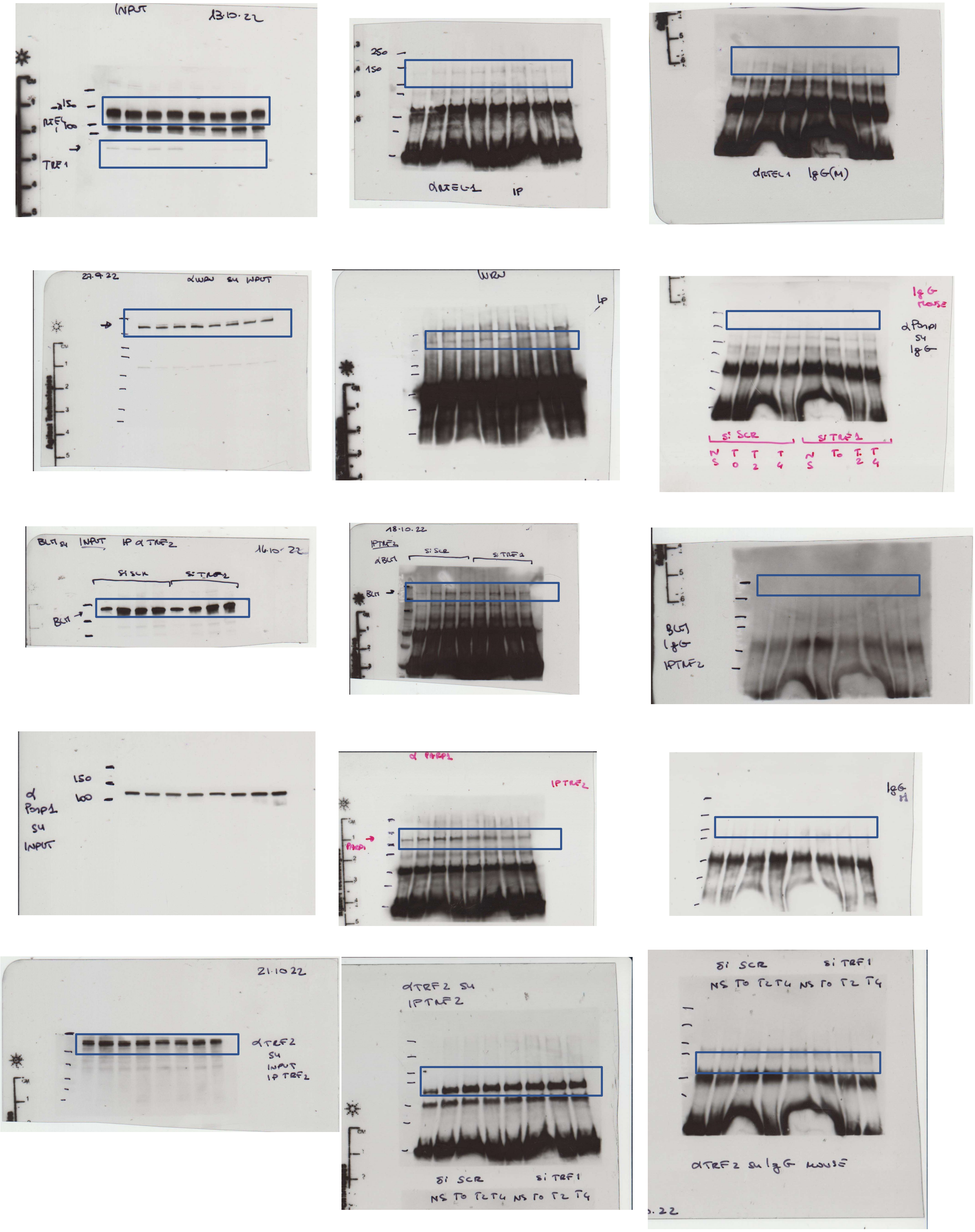

Supplemental figure 12 a

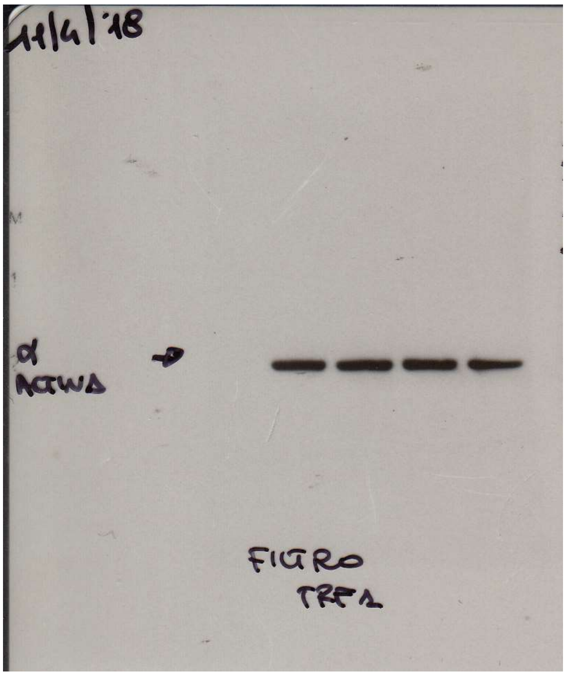

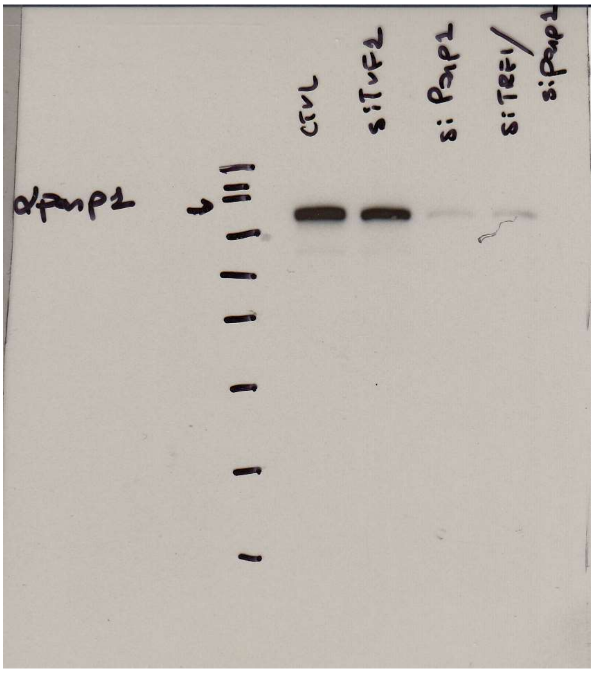

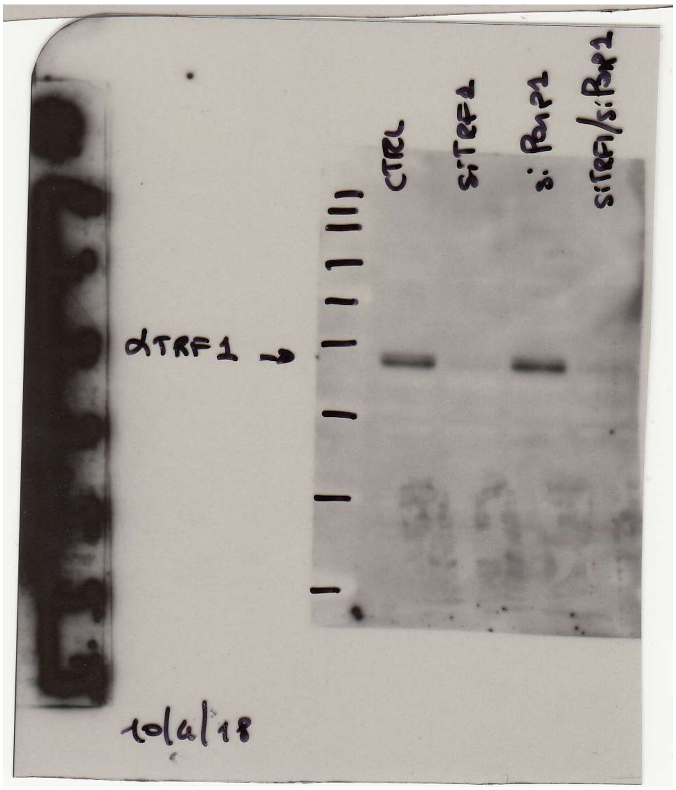

Supplemental figure 13 a

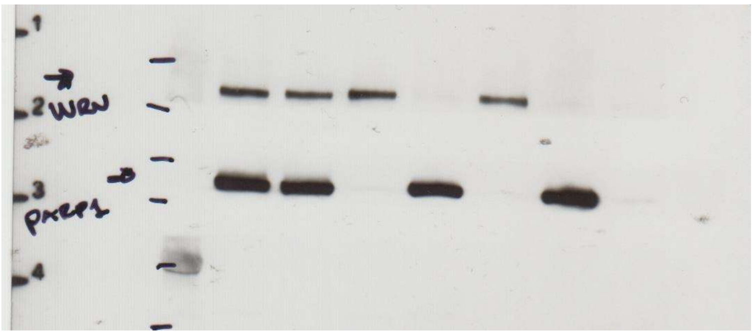

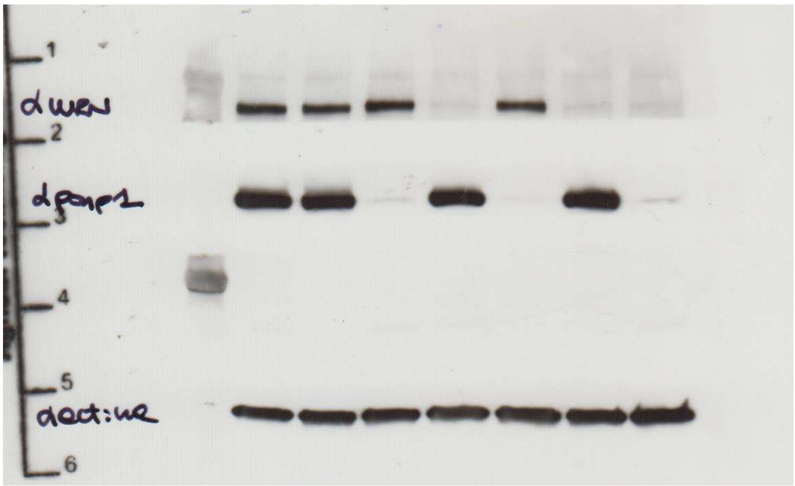

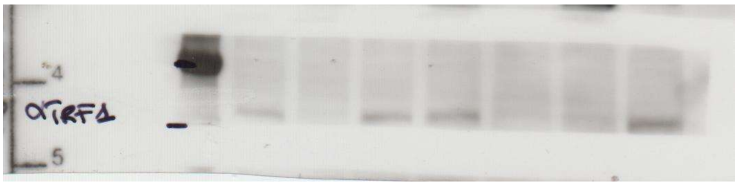

Supplemental figure 15 b

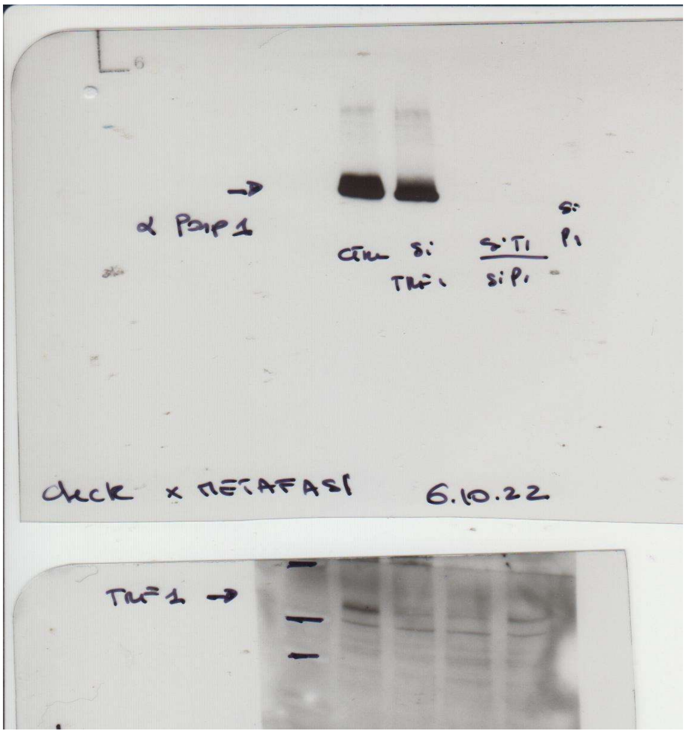

actin

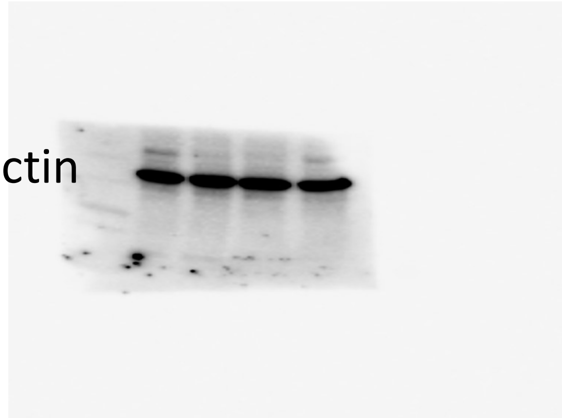

Supplement: Supplementary file 2 — Supplemental figures [file 42003_2023_4596_MOESM2_ESM.pdf]
