# Supplementary material for: PARP1 allows proper telomere replication through TRF1 poly (ADP-ribosyl)ation and helicase recruitment
Source: Commun Biol. 2023 Mar 2;6:234. doi: 10.1038/s42003-023-04596-6 (PMC9981704; doi:10.1038/s42003-023-04596-6)
Supplement: Supplementary file 3 — Description of additional supplementary files [file 42003_2023_4596_MOESM3_ESM.pdf]

Description of Additional Supplementary Files

File Name: Supplementary Data 1

Description: Numerical Source data

File Name: Supplementary Data 2

Description: Numerical Source data
