# Supplementary material for: PARP1 allows proper telomere replication through TRF1 poly (ADP-ribosyl)ation and helicase recruitment
Source: Commun Biol. 2023 Mar 2;6:234. doi: 10.1038/s42003-023-04596-6 (PMC9981704; doi:10.1038/s42003-023-04596-6)
Supplement: Supplementary file 6 — Reporting summary [file 42003_2023_4596_MOESM6_ESM.pdf]

Corresponding author(s): Erica Salvati and Annamaria Biroccio

Last updated by author(s): Feb 14, 2023

Reporting Summary

Nature Portfolio wishes to improve the reproducibility of the work that we publish. This form provides structure for consistency and transparency in reporting. For further information on Nature Portfolio policies, see our Editorial Policies and the Editorial Policy Checklist.

Statistics

For all statistical analyses, confirm that the following items are present in the figure legend, table legend, main text, or Methods section.

n/a	Confirmed	
☐	☒	The exact sample size (*n*) for each experimental group/condition, given as a discrete number and unit of measurement
☐	☒	A statement on whether measurements were taken from distinct samples or whether the same sample was measured repeatedly
☐	☒	The statistical test(s) used AND whether they are one- or two-sided<br>*Only common tests should be described solely by name; describe more complex techniques in the Methods section.*
☒	☐	A description of all covariates tested
☒	☐	A description of any assumptions or corrections, such as tests of normality and adjustment for multiple comparisons
☐	☒	A full description of the statistical parameters including central tendency (e.g. means) or other basic estimates (e.g. regression coefficient) AND variation (e.g. standard deviation) or associated estimates of uncertainty (e.g. confidence intervals)
☐	☒	For null hypothesis testing, the test statistic (e.g. *F*, *t*, *r*) with confidence intervals, effect sizes, degrees of freedom and *P* value noted<br>*Give P values as exact values whenever suitable.*
☒	☐	For Bayesian analysis, information on the choice of priors and Markov chain Monte Carlo settings
☒	☐	For hierarchical and complex designs, identification of the appropriate level for tests and full reporting of outcomes
☒	☐	Estimates of effect sizes (e.g. Cohen's *d*, Pearson's *r*), indicating how they were calculated

Our web collection on statistics for biologists contains articles on many of the points above.

Software and code

Policy information about availability of computer code

Data collection	LASX (Leica), Nis (Nikon) for IF analysis Quantity one (BioRad) chemidoc and phosphoimage acquisition
Data analysis	Microsoft Office 365 (Power Point, Word, Excel) free release for academic Sapienza University, Graphpad Prism 9,Adobe Photoshop CC2018, Image J (free release), Biorender

For manuscripts utilizing custom algorithms or software that are central to the research but not yet described in published literature, software must be made available to editors and reviewers. We strongly encourage code deposition in a community repository (e.g. GitHub). See the Nature Portfolio guidelines for submitting code & software for further information.

Data

Policy information about availability of data

All manuscripts must include a data availability statement. This statement should provide the following information, where applicable:

- Accession codes, unique identifiers, or web links for publicly available datasets
- A description of any restrictions on data availability
- For clinical datasets or third party data, please ensure that the statement adheres to our policy

Raw data are accessible at this site https://gbox.garr.it/garrbox/index.php/s/LJjfkvEA0tvYsf8

Human research participants

Policy information about studies involving human research participants and Sex and Gender in Research.

Reporting on sex and gender	*Use the terms sex (biological attribute) and gender (shaped by social and cultural circumstances) carefully in order to avoid confusing both terms. Indicate if findings apply to only one sex or gender; describe whether sex and gender were considered in study design whether sex and/or gender was determined based on self-reporting or assigned and methods used. Provide in the source data disaggregated sex and gender data where this information has been collected, and consent has been obtained for sharing of individual-level data; provide overall numbers in this Reporting Summary. Please state if this information has not been collected. Report sex- and gender-based analyses where performed, justify reasons for lack of sex- and gender-based analysis.*
Population characteristics	*Describe the covariate-relevant population characteristics of the human research participants (e.g. age, genotypic information, past and current diagnosis and treatment categories). If you filled out the behavioural & social sciences study design questions and have nothing to add here, write "See above."*
Recruitment	*Describe how participants were recruited. Outline any potential self-selection bias or other biases that may be present and how these are likely to impact results.*
Ethics oversight	*Identify the organization(s) that approved the study protocol.*

Note that full information on the approval of the study protocol must also be provided in the manuscript.

Field-specific reporting

Please select the one below that is the best fit for your research. If you are not sure, read the appropriate sections before making your selection.

☒ Life sciences ☐ Behavioural & social sciences ☐ Ecological, evolutionary & environmental sciences

For a reference copy of the document with all sections, see nature.com/documents/nr-reporting-summary-flat.pdf

Life sciences study design

All studies must disclose on these points even when the disclosure is negative.

Sample size	100 metaphase was analysed for telomere fragility assay<br>For IF analysis 100 nuclei/samples were analyzed<br>the sample size was calculated on the basis of previous analysis
Data exclusions	no data were excluded from the analysis
Replication	experiments were replicated three times. Mean and SD are reported. Significant differences were assessed by unpaired Student T test
Randomization	randomization was not relevant to this study since experiments were performed with cultured cell lines which are homogeneous samples
Blinding	investigators were blinded during immunofluorescence and cytogenetic data collection and analysis. in the other assays blind analysis was not applicable

Reporting for specific materials, systems and methods

We require information from authors about some types of materials, experimental systems and methods used in many studies. Here, indicate whether each material, system or method listed is relevant to your study. If you are not sure if a list item applies to your research, read the appropriate section before selecting a response.

Materials & experimental systems

n/a	Involved in the study	
☐	☒	Antibodies
☐	☒	Eukaryotic cell lines
☒	☐	Palaeontology and archaeology
☒	☐	Animals and other organisms
☒	☐	Clinical data
☒	☐	Dual use research of concern

Methods

n/a	Involved in the study	
☒	☐	ChIP-seq
☐	☒	Flow cytometry
☒	☐	MRI-based neuroimaging

Antibodies

Antibodies used	All the antibodies used are specified in the materials and methods included catalog number, clone and manufacturers' data
Validation	Primary antibodies have been validated by RNA interference in PLA or WB (PARP1, TRF1 and WRN) or by reference of the manufacturers

Eukaryotic cell lines

Policy information about cell lines and Sex and Gender in Research

Cell line source(s)	ATCC
Authentication	Since the cells were purchased from repository they were not further authenticated
Mycoplasma contamination	All the cell lines are tested monthly for mycoplasma with N-garde kit Euroclone
Commonly misidentified lines (See ICLAC register)	none

Flow Cytometry

Plots

Confirm that:

☒ The axis labels state the marker and fluorochrome used (e.g. CD4-FITC).

☒ The axis scales are clearly visible. Include numbers along axes only for bottom left plot of group (a 'group' is an analysis of identical markers).

☒ All plots are contour plots with outliers or pseudocolor plots.

☒ A numerical value for number of cells or percentage (with statistics) is provided.

Methodology

Sample preparation	After culturing and treatment, cells were harvested, washed with PBS twice, fixed in 70% ethanol at 4 °C overnight. Then, cells were washed with PBS twice, stained with PI at a final concentration of 50 μg/mL and RNase at a final concentration of 75 kU/mL, incubated for 30 min, then analyzed by FACSCalibur and FACSCelesta (BD Biosciences, San Jose, CA, USA). Progression of cells through the cell cycle phases was analyzed by simultaneous flow cytometric measurements of DNA and 5-bromo-2′-deoxyuridine (BrdU) contents of cells, as previously described [18]. Briefly, cells were pulsed with BrdU (Sigma Aldrich) at a final concentration of 20 μM for 15 min, and after the appropriate intervals in BrdU-free medium (from 2.5 to 24 h) the DNA was denatured. Cells were then incubated with 20 μl of the mouse Mab-BrdU (347580 Pure BD) for 1 hr at room temperature, and BrdU-labeled cells were detected using goat anti-Mouse Fab′2 Alexa Fluor 488 (Cell Signaling). The cells were counterstained with PI, acquired and analyzed.
Instrument	BD FACS Calibur Nr. 10465, BD FACS Celesta Nr. R66034400279
Software	BD Cellquest, BD Diva, BD ModFit
Cell population abundance	20000 events/sample were acquired at least
Gating strategy	During data acquisition a threshold trigger and FSC/SSC parameters has been used to gate out cellular debris. Cell clumps or doublets have been eliminated by using Doublet Discrimination Module (DDM) of FL3-A versus FL3-W. Boundaries between positive and negative samples have been indicated as a black line in figure 1C and 4A

☐ Tick this box to confirm that a figure exemplifying the gating strategy is provided in the Supplementary Information.
